# Strategies for simultaneous strengthening and toughening via nanoscopic intracrystalline defects in a biogenic ceramic

**DOI:** 10.1038/s41467-020-19416-2

**Published:** 2020-11-10

**Authors:** Zhifei Deng, Hongshun Chen, Ting Yang, Zian Jia, James C. Weaver, Pavel D. Shevchenko, Francesco De Carlo, Reza Mirzaeifar, Ling Li

**Affiliations:** 1 Department of Mechanical Engineering, Virginia Polytechnic Institute and State University, 635 Prices Fork Road, Blacksburg, VA 24061 USA; 2 John A. Paulson School of Engineering and Applied Sciences, Harvard University, 60 Oxford Street, Cambridge, MA 02138 USA; 3 Advanced Photon Source, Argonne National Laboratory, 9700S Cass Ave, Lemont, IL 60439 USA

**Keywords:** Biomineralization, Mechanical properties

## Abstract

While many organisms synthesize robust skeletal composites consisting of spatially discrete organic and mineral (ceramic) phases, the intrinsic mechanical properties of the mineral phases are poorly understood. Using the shell of the marine bivalve *Atrina rigida* as a model system, and through a combination of multiscale structural and mechanical characterization in conjunction with theoretical and computational modeling, we uncover the underlying mechanical roles of a ubiquitous structural motif in biogenic calcite, their nanoscopic intracrystalline defects. These nanoscopic defects not only suppress the soft yielding of pure calcite through the classical precipitation strengthening mechanism, but also enhance energy dissipation through controlled nano- and micro-fracture, where the defects’ size, geometry, orientation, and distribution facilitate and guide crack initialization and propagation. These nano- and micro-scale cracks are further confined by larger scale intercrystalline organic interfaces, enabling further improved damage tolerance.

## Abstract (web summary)

Biominerals are nanocomposites that often incorporate nanoscopic defects such as organic inclusions within the mineral matrix. Here, the authors report on an experimental and computational study into the effects of intracrystalline defects on the intrinsic mechanical behaviour of biominerals.

## Introduction

Skeletal materials in biological systems are typically composites that consist of mineral and organic phases^1–7^. The biomineralized building blocks are often hierarchically organized across multiple length scales in these composites, which is responsible for their remarkable mechanical performance^8^. In contrast to pure geological or synthetic minerals, those formed through biologically controlled processes usually exhibit nanoscopic structural heterogeneities through the incorporation of intracrystalline macromolecules within their crystalline mineral matrices^9–12^. The intracrystalline organic matter in the extensively studied mollusk shells have been shown to primarily consist of proteinaceous materials^13–16^. These intracrystalline proteins often include a high percentage of acidic amino acids, such as aspartic acid, which can be secondarily modified by sugar residues^9^. Previous studies have shown that the proteins extracted from biogenic minerals can induce changes in crystal morphology during the in vitro formation process of minerals^13,17–19^. In particular, it has been suggested that the highly acidic groups preferentially bind to calcium ions or the (0001) planes in calcite^20,21^, where stereochemical effects may also contribute to this process^13^. Moreover, the incorporation of intracrystalline organic inclusions is proposed to be the main structural origin for the lattice distortions in biogenic crystals, which is widely observed in many biogenic calcium carbonate systems^22–24^.

Although the importance of the hierarchical designs in biological composites is well recognized in the literature, the intrinsic mechanical properties of individual mineral building blocks are yet to be fully elucidated. The mechanical strengthening effects of the intracrystalline defects have been suggested through the mechanical characterization of biomimetic synthetic minerals with incorporated amino acids, micelles, or other entities^25–27^. However, the relationship between the morphology of these intracrystalline defects (e.g., size, spacing, geometry, orientation, and distribution) and the deformation behavior of biominerals, especially in the inelastic regime, is yet to be established. For example, the “conchoidal” fracture behavior of biogenic calcite, one of the most abundant biominerals, has long been recognized despite its unclear mechanisms, and represents one of the most intriguing puzzles in the field of biomineralization^11,28^. Beyond its biological implications, understanding the intrinsic mechanical properties of biominerals is also technically important for the design and synthesis of biomimetic structural materials with improved mechanical performance^29–31^.

Direct measurement of the intrinsic mechanical properties of biogenic minerals in these skeletal composites, however, is challenging due to their complex hierarchical organization, which often prevents effective isolation of individual units and precise control of the loading and boundary conditions during mechanical characterizations. Here we approach this problem by studying the intrinsic mechanical behavior of biogenic calcite from the bivalve *Atrina rigida* through quantitative multiscale mechanical characterization, systematic structural analysis before and after deformation, and theoretical and computational modeling. The results reported here elucidate the fundamental mechanical roles of the nanoscopic intracrystalline defects in these calcitic biominerals, including strengthening by suppressing dislocation slips and toughening by promoting fracture along non-cleavage lattice planes. These small-scale toughening mechanisms are further amplified by structural hierarchies at larger length scales for additional damage localization and energy dissipation.

## Results

### Hierarchical structure of the biogenic calcite in *Atrina rigida*

**Fig. 1:**
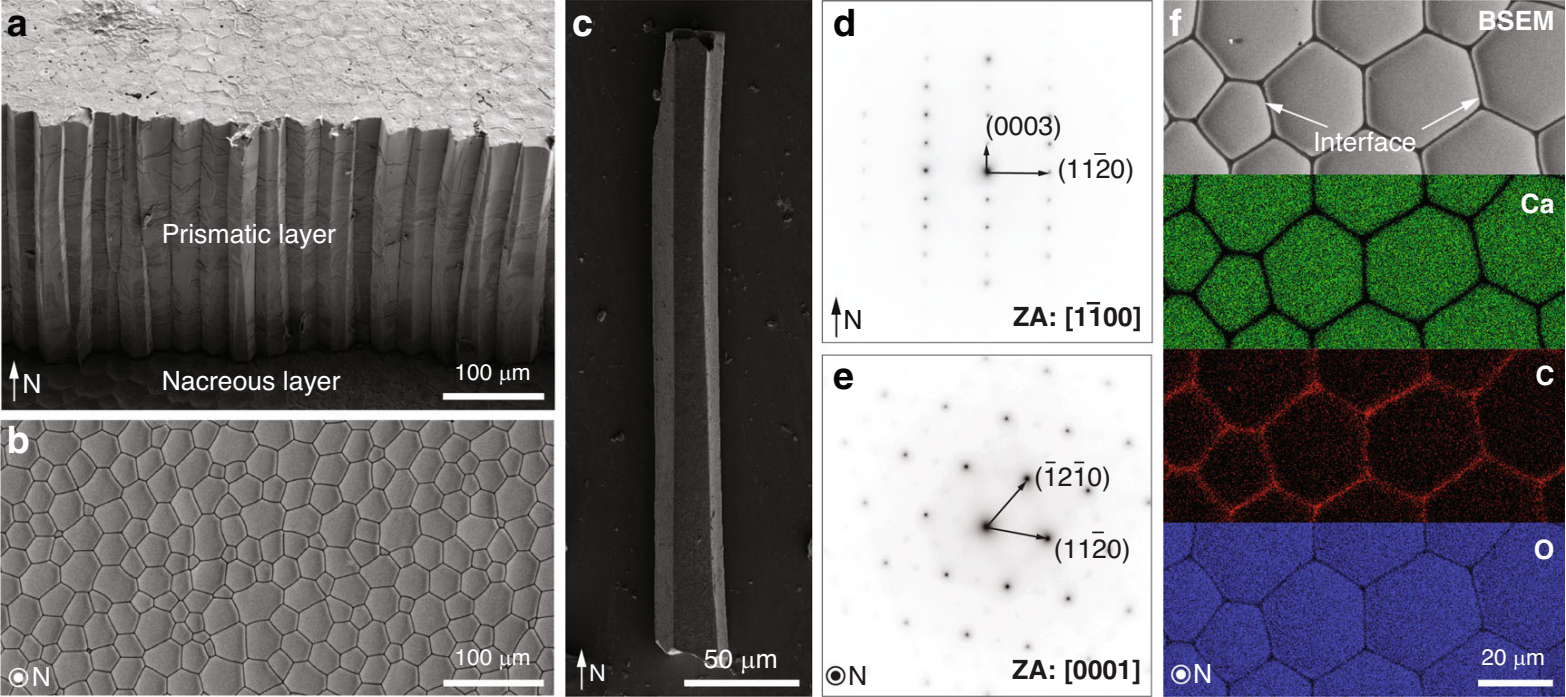
Biogenic calcite in the prismatic layer of the mollusk *Atrina rigida*. **a** Scanning electron micrograph (SEM) of the shell cross-section showing the prismatic layer (upper region) and the nacreous layer (lower region) in the shell. The normal direction of the shell is indicated by the arrow (“N”). **b** Backscattered SEM (BSEM) image of the prismatic layer sectioned and polished in the direction perpendicular to “N”, which shows the polygonal cross sections of the prismatic crystals. **c** SEM image of one isolated prismatic crystal. **d**, **e** Selected area electron diffraction (SAED) patterns taken from two sections prepared from two orientations, **d** parallel to the normal direction (zone axis (ZA): [1$\bar 1$00]) and **e** perpendicular to the normal direction (ZA: [0001]). **f** Energy dispersive spectroscopy (EDS) elemental mapping of the calcitic prisms and their intercrystalline organic interfaces. From top to the bottom, BSEM image revealing the local differences in electron density, and their corresponding Calcium, Carbon, and Oxygen maps.

The pen shell, *A. rigida*, is a bivalve mollusk native to Caribbean Sea, and has shells consisting of two distinct mineral phases, an inner aragonitic nacreous layer and an outer calcitic prismatic layer (Fig. 1a). The inner nacreous layer has the well-known “brick-and-mortar” structure, while the outer prismatic layer consists of prism-like calcite crystals with polygonal cross sections (Fig. 1b)^16,32^. These prismatic calcite crystals typically measure 20–50 μm in diameter and several hundreds of micrometers in length along the shell normal (N direction, Fig. 1b, c). Selected area electron diffraction (SAED) patterns acquired from these calcitic prisms reveal their single crystal nature (Fig. 1d, e), with their *c*-axes generally aligned with their longitudinal axis^10,11^. At the boundaries between adjacent calcite prisms are thin intercrystalline organic layers (thickness: *ca.* 500 nm) (Fig. 1f)^16,33^, which consist of glycine-rich proteins and are believed to provide a scaffold for the templated growth of the calcitic prisms^16^.

**Fig. 2:**
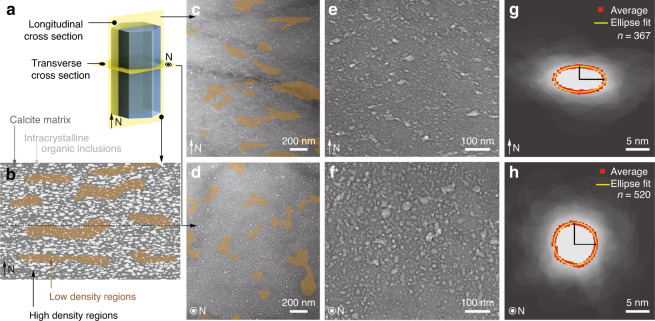
Structural characterizations of the intracrystalline inclusions in the prisms of *Atrina rigida*. **a** Illustration of sample orientations (parallel and perpendicular to N) for analysis using transmission electron microscopy (TEM) . **b** Schematic diagram illustrating the presence and distribution of low electron density intracrystalline defects when viewed in the direction parallel to N. **c**, **d** Low-magnification and **e**, **f** high-magnification TEM images of the biogenic calcite, revealing regions of high and low density of defects (highlight with brown shaded areas) in two orientations: **c**, **e** parallel to and **d**, **f** perpendicular to the normal direction. **g**, **h** Images of overlaid contours of individual intracrystalline defects in two orientations: **g** parallel to and **h** perpendicular to the normal direction. The red dotted and yellow solid lines are the average and fitted profiles, respectively.

In addition to the intercrystalline organic interfaces, defect-like nanoscopic inclusions, which were previously demonstrated to contain organic materials^10^, are present within the single-crystal calcite prisms. These defects can be visualized with bright-field transmission electron microscopy (TEM) imaging due to their comparatively low electron densities (Fig. 2a–f and Supplementary Fig. 1). While previous studies estimated the presence of *ca*. 0.4 wt% of intracrystalline organic material within these calcite prisms^16^, the distribution of these defects is not homogeneous. Instead, the defects exhibit regions with high and low densities that are clearly visible in low-magnification TEM images (Fig. 2b–d). When viewed in longitudinal cross section, the high- and low-density regions exhibit an alternating zone-like morphology, with a characteristic spacing of several hundreds of nanometers (Fig. 2b, c). Etching experiments performed on the longitudinally polished sections also reveal this pseudo-layered structure within the calcitic prisms (Supplementary Fig. 2). This modulation in the distribution of intracrystalline defects was proposed to be related to their sequential stepwise deposition during the process of shell growth^16^. For comparison, regions with high- and low-densities of intracrystalline defects are randomly distributed in the transverse cross section (Fig. 2d). Moreover, individual defects are elongated along the horizontal direction when viewed in the longitudinal cross section (Fig. 2e), but circular in the transverse cross-sections (Fig. 2f), consistent with previous studies^10^. Quantitative geometrical measurements show that these defects can be represented as equal-axed ellipsoids with a height of *ca*. 5 nm and a lateral span of *ca*. 10 nm (Fig. 2g, h and Supplementary Note 1 and Supplementary Fig. 3).

### Uniaxial micro-pillar compression tests

**Fig. 3:**
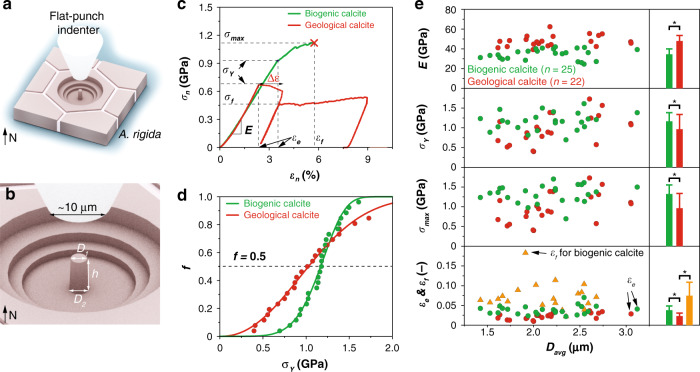
Quantitative mechanical properties of biogenic and geological calcite via uniaxial micro-pillar compression tests. **a**, **b** Schematic illustrations of the uniaxial compression of micro-pillars with a flat-end diamond tip (bottom diameter, 10 µm). The parameters of the micro-pillars include *D*_1_ (top diameter), *D*_2_ (bottom diameter), and *h* (height of the micro-pillars). **c** Representative normalized engineering stress-strain (*σ*_*n*_*-ε*_*n*_) curves for uniaxial compression of the biogenic and geological calcite micro-pillars. The calculated mechanical parameters include *E* (Young’s modulus), *σ*_*Y*_ (yield strength), *σ*_max_ (maximum strength), *ε*_*e*_ (elastic limit), *ε*_*f*_ (failure strain), and *σ*_*f*_ (flow strength for geological calcite). **d** Weibull analysis of *σ*_*Y*_, where *f* is the failure percentage. **e** Measured distribution of *E*, *σ*_*Y*_, *σ*_max_, *ε*_*e*_, and *ε*_*f*_ as functions of the pillar average diameter, *D*_avg_. The histograms on the right show the average and standard deviation of each data set. Asterisks represent statistical significance at a level of 0.05 via two sample *t*-tests.

**Fig. 4:**
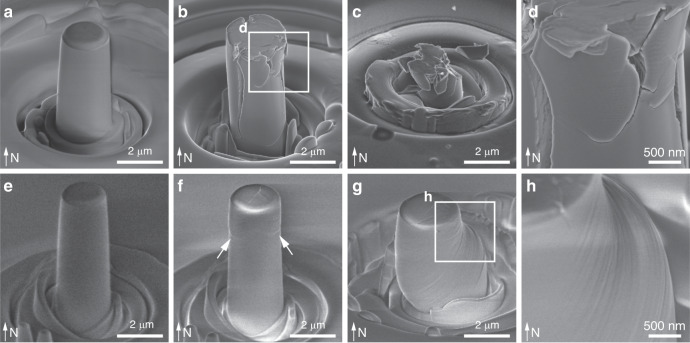
Deformation of biogenic and geological calcite via uniaxial micro-pillar compression tests. SEM images of **a**–**d** biogenic and **e**–**h** geological calcite under sequential deformation steps, including the calcite **a**, **e** before compression, **b**, **f** immediately after yielding, and **c** after complete fracturing or **g** significant plastic deformation. White arrows in **f** show the initial slip-induced deformation in geological calcite. **d**, **h** High-magnification SEM images show **d** microcracking in the *A. rigida* biogenic calcite, and **h** slips in the geological calcite, respectively.

To probe the intrinsic mechanical properties of the biogenic calcite in *A. rigida*, we performed microscopic uniaxial compression tests on micro-pillars fabricated via focused ion beam (FIB) milling from individual prismatic crystals (Figs. 3a and 4a). Micro-pillars of both biogenic and geological (single crystal Icelandic spar) calcite were prepared with their *c*-axes in the vertical direction, with typical dimensions of *D*_1_ (top diameter) *ca*. 2 μm, *η* (aspect ratio) *ca*. 3, and *α* (taper angle) *ca*. 2.5° (Fig. 3b). The as-measured force, *F*, and compression depth, *δ*, were converted to normalized engineering stress, *σ*_*n*_, and strain, *ε*_*n*_, by applying Sneddon’s formula, which accounts for the substrate compliance for a slightly tapered pillar^34^. Typical *σ*_*n*_-*ε*_*n*_ data obtained under displacement control conditions (displacement rate: 10 nm/s) for both the biogenic and geological calcite are shown in Fig. 3c (see full data in Supplementary Fig. 5). Both materials exhibited a linear elastic regime before yielding, and the Young’s moduli, *E*, were measured to be 34.4 ± 5.3 (average ± standard deviation, *n* = 25) and 47.9 ± 5.5 GPa (*n* = 22) for the biogenic and geological calcite, respectively (Fig. 3e). The *A. rigida* biogenic calcite exhibited a slightly higher yield strength, *σ*_*Y*_, in comparison to its geological counterpart (1.16 ± 0.22 vs. 0.96 ± 0.37 GPa, Fig. 3e), which is consistent with the increase of hardness measured from instrumented nanoindentation (Supplementary Fig. 6). Moreover, the Weibull analysis suggests that the *A. rigida* biogenic calcite has a smaller variation for *σ*_*Y*_ as indicated by its higher Weibull modulus (*A. rigida* biogenic calcite: 5.74 ± 0.30; geological calcite: 2.24 ± 0.06, Fig. 3d). The *A. rigida* biogenic calcite also yielded at a higher strain level as indicated by its greater elastic limit, *ε*_*e*_, (*A. rigida* biogenic calcite: 0.0379 ± 0.0109; geological calcite: 0.0229 ± 0.0077, Fig. 3e).

The micro-compression tests also revealed dramatically different inelastic deformation behavior between the *A. rigida* biogenic and geological calcite. The *A. rigida* biogenic calcite continued to carry load after yielding as indicated by the gradual increase of *σ*_*n*_ with *ε*_*n*_, leading to a failure strain (*ε*_*f*_) of 0.0740 ± 0.0341 until an abrupt fracture (Fig. 3c). This behavior also resulted in an ultimate fracture strength (*σ*_max_) of 1.32 ± 0.23 GPa for the *A. rigida* biogenic calcite (Fig. 3e). Inspection of compression test results performed on biogenic calcite, which were stopped before final failure, revealed the presence of micro-cracks running vertically, which were often later deflected horizontally (Fig. 4b, d). Surprisingly, the damaged biogenic calcite could still sustain load and eventually fractured into microscopic pieces (Fig. 4c). Geological calcite, on the other hand, exhibited a large displacement jump accompanied by an appreciable load drop at the yield point (Fig. 3c). After the compressive loading was re-established, it yielded at smaller stresses followed by a steady plastic flow (*σ*_*f*_, 0.47 ± 0.12 GPa, *n* = 14). Geological calcite micro-pillars exhibited first permanent deformation in their upper regions  during compression tests, as evidenced in the samples where loading was immediately stopped after yielding (Fig. 4f), while those after significant inelastic deformation showed classical slip steps on the surface of the micro-pillars (Fig. 4g, h). Such behavior has been observed in slip events or dislocation avalanches of some metals during displacement-controlled compression, which leads to a sudden increase in the displacement (Δ*ε*) at the yield point (Fig. 3c), followed by an “unloading” segment, during which the compression device retracts the tip and then a re-loading segment after the displacement matches the pre-set time-displacement profile^35^. Once the stress level reached the yield point, the geological calcite micro-pillar underwent a steady plastic flow regime and significant permanent deformation was observed (Fig. 4g, h). Unlike the relatively uniform stress field generated by uniaxial compression, indentation induces a complex three-dimensional stress field, under which the geological calcite is more prone to fracture, evidenced by its larger fracture pattern size in comparison to the *A. rigida* biogenic calcite (Supplementary Fig. 7).

### Microscale deformation mechanisms

**Fig. 5:**
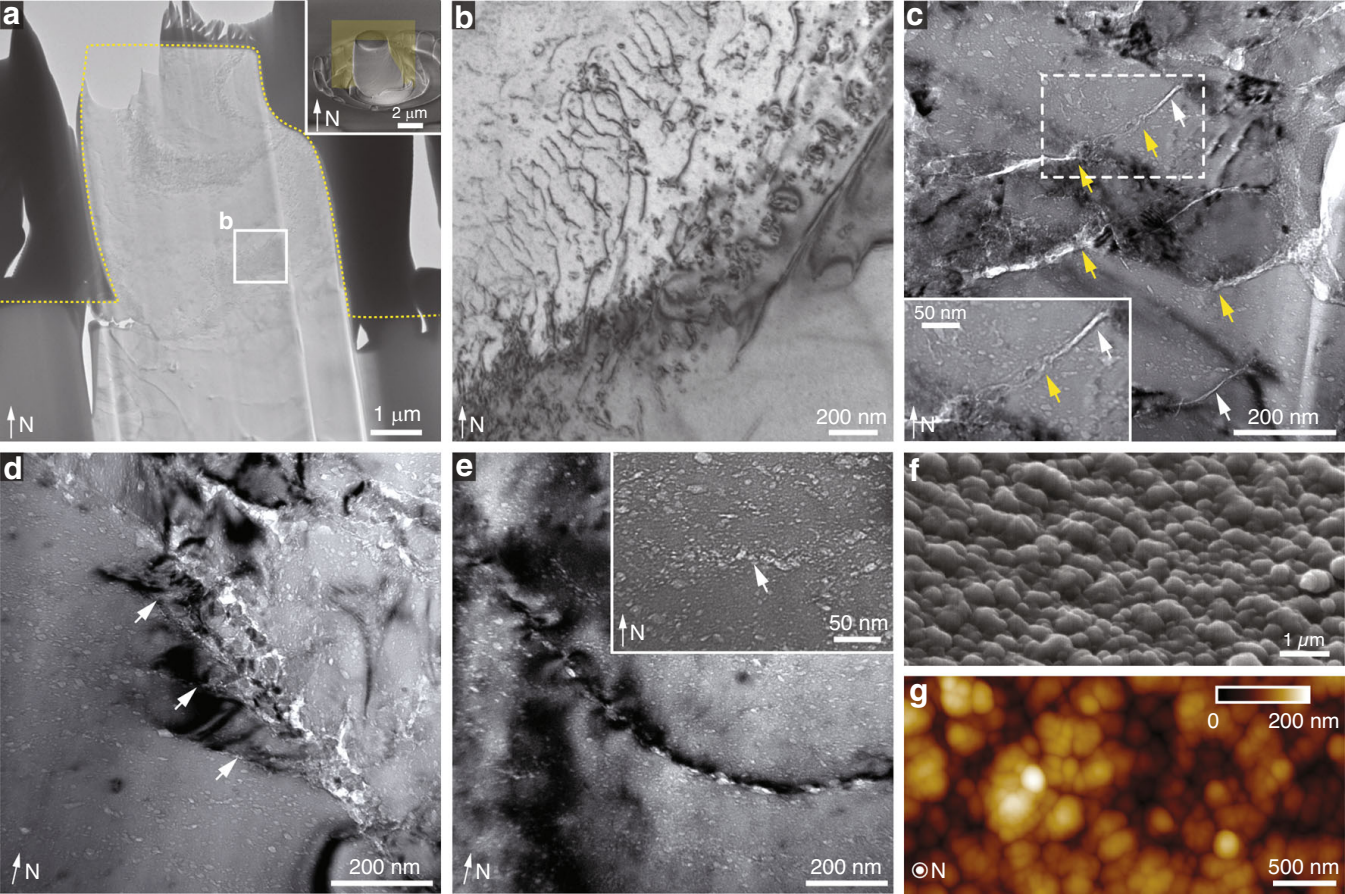
Microscale deformation mechanisms in biogenic and geological calcite. **a** TEM image of a thin slice taken from a significantly deformed geological calcite micro-pillar after compression testing. Inset, SEM image of the micro-pillar used for TEM sample preparation and the yellow plane shows the orientation of the TEM slice. **b** High magnification TEM image showing the dislocation arrays developed within the deformed geological calcite micro-pillar. **c** TEM image showing micro-/nano-scale cracks developed within the *A. rigida* biogenic calcite after deformation. White and yellow arrows indicate the cracks in the low- and high-density defect regions, respectively. **d** TEM image taken at the boundary of a fractured zone in biogenic calcite, showing the propagation directions of several cracks (white arrows) in parallel to the long axes of the defects, which is perpendicular to the calcite’s *c*-axis. **e** TEM image demonstrating that a crack formed via the coalescence of micro-cracks initiated from defects and followed the long-axis directions of defects. Inset: TEM imaging showing a site of crack initiation through nanoscopic damage coalescence from adjacent defects. **f** SEM and **g** atomic force micrograph (AFM) of the fractured surface of biogenic calcite, showing the nanoscale rough surfaces.

Thin sections prepared from micro-pillars after compression tests were used for TEM imaging to further understand the underlying deformation mechanisms (Fig. 5). For micro-pillars of geological calcite after significant plastic deformation, a large number of dislocations were generated, where individual dislocation lines are highly curved without well-defined geometries, a characteristic of dislocations in calcite (Fig. 5a, b)^36^. It has long been known that geological calcite deforms plastically via deformation twinning and slip, particularly at elevated temperatures and confined hydrostatic pressures^37–39^. The primary plastic deformation mechanisms in geological calcite include twinning on *e* {$\bar 1$018}<40$\bar 4$1>^+^ (three systems), slip on *r* {10$\bar 1$4}<$\bar 2$021>^±^ (three systems), and slip on *f* {$\bar 1$012}<2$\bar 2$01>^±^ (six systems)^36,39^. In the present study, uniaxial compression of the calcite micro-pillars along the *c* axis led to the highest Schmid factors (*m*) in the *r* systems (0.49996), resulting in the activation of this slip system in the negative sense (Supplementary Note 3), which was also confirmed through the direct comparison of the slip morphology between experimental observations and structural modeling (Supplementary Fig. 8). Gliding on two and sometimes on all three equivalent *r* systems led to retention of the circular cross section after compression (Supplementary Figs. 9 and 10 and Supplementary Note 4). In our testing conditions, we did not observe significant slips in *e* or *f* systems, or *e*-twining. TEM analysis of micro-pillars immediately after yielding revealed that dislocations were concentrated in the top portion of micro-pillars, consistent with SEM observations (Fig. 4f and Supplementary Fig. 11).

In stark contrast to geological calcite, TEM imaging of the *A. rigida* biogenic calcite after deformation revealed that micro-cracking, instead of dislocation slipping, was the dominating deformation mechanism (Fig. 5c–e). More importantly, these cracks are more tortuous in the regions with higher densities of intracrystalline defects (yellow arrows in Fig. 5c). This behavior was also evident from TEM imaging of a thin section before and after crack formation, revealing that cracks were deflected at the nanoscale by defects, particularly in high-density regions (Supplementary Fig. 12). Additionally, these microcracks tended to propagate along the prisms’ transverse orientation, which is parallel to the intracrystalline defects’ long-axis directions (white arrows in Fig. 5d). This fracture behavior leads to the formation of layered pieces in some fracture surfaces (Supplementary Fig. 13). The thickness of these layered pieces is approximately several hundred nanometers, matching well with the characteristic length scale in the density modulation of intracrystalline defects along the *c*-axis.

In addition to regulating crack propagation, the intracrystalline defects also affect the initial formation of micro-cracks. Figure 5e shows a forming crack as it travels through a series of adjacent intracrystalline defects along their long axes over a distance of several hundred nanometers. The inset shows the formation of a crack embryo in a region with high defect density, which just begun to bridge adjacent defects (white arrow). Similar crack initiation processes were also observed in our indentation samples (Supplementary Fig. 14).

The cracking pattern facilitated by the intracrystalline defects produced rough fracture surfaces with nanoscopic bumps of typical height and lateral spans on the order of several hundred nanometers (Fig. 5f, g), which is on the same length scale as that of the heterogeneous distribution of intracrystalline defects. Results obtained from in situ SEM mechanical experiments revealed fracture surfaces with similar morphologies, thus eliminating the possibility that the observed features were artifacts from the employed sample preparation protocols (Supplementary Fig. 15). Regions with a low-density distribution of defects exhibited a similar fracture pattern to those seen in geological calcite, i.e., cleavage along {10$\bar 1$4} planes (white arrows in Fig. 5c and Supplementary Fig. 12).

**Fig. 6:**
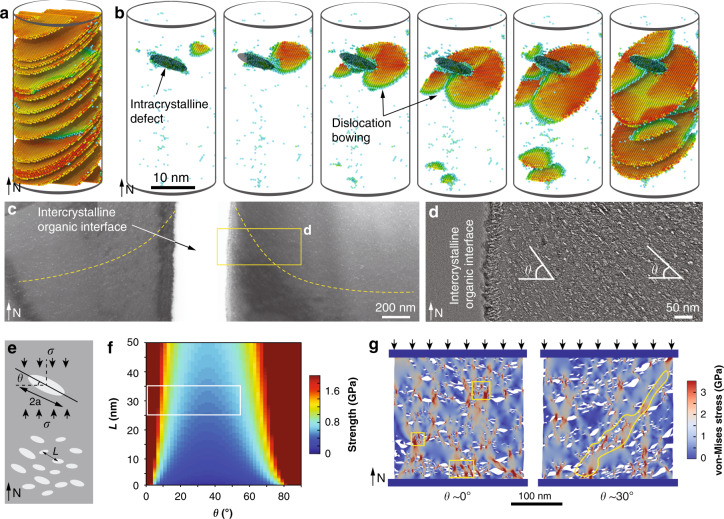
Mechanical effects of intracrystalline defects in *A. rigida* ﻿biogenic calcite. **a**, **b** Atomistic simulation of compression on **a** geological and **b** the biogenic calcite along the *c* axis, respectively. **c**, **d** TEM images showing that the orientation of the intracrystalline defects is gradually tilted towards the intercrystalline organic interfaces. The yellow dashed lines in **c** indicate the long-axis direction of defects locally. **e** Schematic model of the biogenic calcite with the presence of intercrystalline defects. **f** Estimated strength in relation to the spacing (*L*) and the inclination angle (*θ*) of intracrystalline defects. The boxed region highlights the actual configurations of defects observed in the *A. rigida* biogenic calcite. **g** Comparison of the stress contours and fracture patterns of biogenic calcites with defects orientations *θ* = *ca.* 0° and *ca.* 30° based on finite element/discrete element simulation. The boxed regions in the *θ* = *ca.* 0° model show cracks initiated at regions with a high density of defects, and the yellow region highlights an inclined damage band formed in the *θ* = *ca.* 30° model.

The increase in strength directly measured from the micro-pillar compression tests is consistent with our results and previous studies that showed higher hardness in various biogenic calcites compared to their geological counterparts^40–42^. Polishchuk et al. recently showed that the biogenic calcite in the brittle star *Ophiocoma wendtii* is strengthened by Mg-rich nanodomains through a compressive stress field similar to Guinier-Preston’s zones in metals^43^. The Mg concentration in the calcite prisms of *A. rigida* (0.36 ± 0.3 at% based on energy dispersive X-ray spectroscopy, consistent with ref. ^44^), however, is much lower than that from brittle stars (3.03 ± 0.04 at%)^43^ and other echinoderms^44–46^. Previous studies have also shown that the incorporation of Mg is likely to contribute only *ca.* 20% of the hardness increase in the biogenic calcite of *A. rigida*^40,44^, suggesting that Mg is unlikely to be the major contributing factor for the increased strength. Moreover, the post-deformation TEM imaging clearly revealed that the dislocation slips observed in geological calcite were effectively suppressed in the *A. rigida* biogenic calcite. These results suggest that the nanoscopic intracrystalline defects strengthen the *A. rigida* biogenic calcite through a strategy similar to the classical precipitation strengthening by restricting dislocation motions^47,48^. This mechanism is further demonstrated with our atomistic modeling, where the propagation of *r*-slips in calcite can be significantly impeded due to the presence of nanoscopic defects (Fig. 6a, b). Unlike dislocation slips where translational motion of atoms along the slip planes are required, deformation-induced twining through the formation of coherent twining boundaries is not impeded by the intracrystalline defects (Supplementary Fig. 16). Such twining boundaries may contribute to increased damage localization and penetration resistance, particularly under indentation loading conditions, as reported recently in other biogenic calcite systems^2^.

### Multiscale toughening mechanisms

The increase of strength (∆*σ*_*Y*_) due to dislocation restriction is estimated as *ca*. 0.36 GPa following the relationship *H* = *H*_0_ + *C*∆*σ*_*Y*_, where *H* and *H*_0_ are the hardness of the *A. rigida* biogenic and geological calcites, respectively, and *C* is a material-specific coefficient (Supplementary Note 5)^49^. This enhancement leads to the estimated strength of *ca*. 1.32 GPa for the *A. rigida* biogenic calcite, which is higher than those measured experimentally (1.16 ± 0.22 GPa). This result suggests that fracture should occur before yielding (dislocation motion) in the *A. rigida* biogenic calcite, consistent with the lack of observed dislocations in fractured *A. rigida* specimens (Fig. 4b–d, compared to Fig. 4g, h). This observation leads to the classical strength-toughness dilemma in structural materials, as fracture is usually considered a less effective path for energy dissipation^50^. For example, the geological calcite exhibits the brittle fracture behavior under indentation and large cleavage cracks are developed (Supplementary Fig. 7)^51^. This material design challenge is solved in the *A. rigida* biogenic calcite by controlling the fracture process through the intracrystalline defects as well as the interaction with higher hierarchical structural features.

**Fig. 7:**
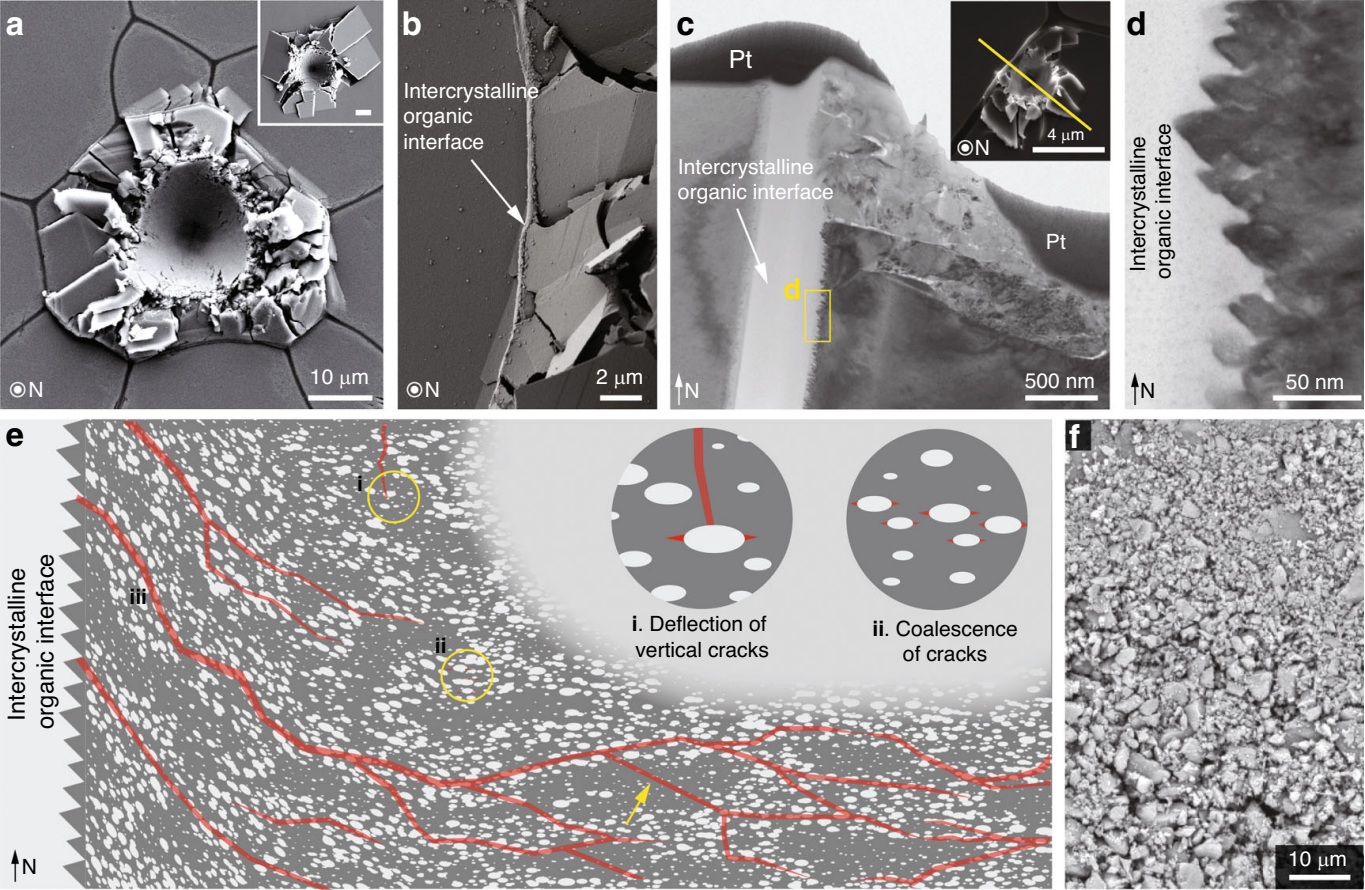
Synergistic toughening mechanisms across multiple length scales. **a** Plan view SEM image of a high-load indentation on *A. rigida* biogenic calcite (conospherical tip; tip radius = 5 μm; semi-angle = 45°; maximum load = 500 mN). Inset: Plan view SEM image of geological calcite under the same loading condition; scale bar 10 μm. **b** Damage localization by the intercrystalline organic interfaces as the micro-fracturing in the prism on the right was completely stopped by the interface. **c** Cross-sectional TEM image showing that the inelastic deformation was effectively localized by the organic interface beneath the sample surface. Inset: Plan view SEM image of the original indentation residue. The yellow solid line indicates the location and orientation of the TEM sample prepared by FIB. Pt, protective platinum layer. **d** A characteristic saw-tooth-like microstructure observed on the vertical surface of the prisms. **e** Schematic illustrating the regulation of crack formation and propagation (indicated by red lines) in the *A. rigida* biogenic calcite due to the intracrystalline defects, including (i) crack deflection; (ii) crack initiation resulting from micro-crack coalescence in regions with high defect density; and (iii) extensive upwardly directed cracks due to the inclined defects near prism boundaries. The yellow arrow shows that regions with a low density of defects fracture similar to pure calcite. **f** Micro-/nano-scale fractured pieces of the *A. rigida* biogenic calcite after extensive deformation.

On the individual defect level, the elliptical geometry of the intracrystalline defects (with their long axes oriented along the transverse direction of the calcite prisms) leads to a more than doubled stress intensity factor along their long axes compared to their short axes (Supplementary Note 6). Moreover, the intracrystalline defects can lead to crack-blunting, or cause a vertically propagating crack to be deflected in a horizontal direction, following the well-known Gordon-Cook effect (Fig. 7e, mechanism i, and Supplementary Note 6)^52^. These combining effects lead to preferred crack propagation along the long axis direction of the defects (i.e. the transverse direction of prisms), as observed experimentally. These effects can be further enhanced by the presence of defects with sharp corners along their long axes (see representative TEM images in Supplementary Fig. 17), which have also been reported in other biominerals^53,54^.

On the individual prismatic crystal level, in addition to the pseudo-layered distribution of regions exhibiting high and low defect density, the intracrystalline defects also exhibit a judicious spatial control in their orientations. More specifically, the long axes of the defects, which are primarily oriented along the transverse direction of the prisms (inclination angle, *θ* < 5°), become gradually more tilted towards the intercrystalline organic interfaces (*θ* > 30°, Fig. 6c, d, Supplementary Note 1 and Supplementary Fig. 4). Following the previous micromechanical model for quasi-brittle materials with pre-existing defects^55^, the fracture strength of the *A. rigida* biogenic calcite (*σ*_*y*_) is governed by the wing crack formation and crack coalescence from intracrystalline defects, which strongly depends on their spacing (*L*) and orientation (*θ*), and *σ*_*y*_ can be evaluated via

1 $$\sigma _y = \left( {\frac{{2\pi }}{{11}}} \right)^{1/4}L^{1/2}\left( {\frac{{\sigma _{Y,c}\sqrt {\pi a} }}{{20\beta (\theta )a}}} \right)$$

where *β*(*θ*) is a function of *θ* and the frictional coefficient between the defects and the matrix, *a* is the half length of the defects (*ca.* 5.5 nm), and *σ*_*Y,c*_ represents the compressive strength of geological calcite (0.96 GPa based on micro-pillar compression results) (Fig. 6e and Supplementary note 7). As shown in Fig. 6f, as the defect spacing *L* reduces from the nominal value of *ca*. 32 nm to 10 nm in the high-density regions, the strength *σ*_*y*_ reduces by *ca*. 0.4 GPa; a similar reduction can be achieved when *θ* increases to 30–50°. Finite element/discrete element-based fracture simulations further confirm that: (1) local damage tends to initiate at regions with a high density of defects through crack coalescence along the long-axis directions of defects (Fig. 6g, and Fig. 7e mechanism ii), which is consistent with TEM observations (Fig. 5e), and (2) regions with higher inclination angles have lower strength by channeling cracks along the alignments of defects (Fig. 7e mechanism iii, Supplementary note 8, and Supplementary Fig. 18). With control of their size, geometry, orientation, spacing, and distribution, these intracrystalline defects effectively control the initiation and propagation of micro-cracks beyond the intrinsic preference of cleavage-plane fracture of pure calcite. It should be noted that this mechanism differs from the prestressing strategy by the coherent Mg-rich nanodomains reported in the calcitic skeleton of brittle stars, which generate local compressive stress fields in the mineral matrix to impede cracking^43^. Moreover, the controlled fracture process via judicial placement of intracrystalline defects within the continuous *A. rigida* biogenic calcite reported here is also distinct from the toughening strategy by the nanogranular features, which are often observed in aragonite-based biomineral building blocks^56^. Previous experimental investigations revealed that this nanograin morphology in aragonitic nacre tablets enhances toughness by facilitating torturous crack propagation through intergranular organic matrix^57^, grain rotation and deformation^58,59^.

The capacity of energy dissipation and damage tolerance in the biogenic calcitic composite of *A. rigida* shells is further enhanced through the combined effect of different levels of its structural hierarchy. For example, while high load indentation (maximum load = 500 mN) induced extensive fracture in the prisms, the damage was well constrained within a localized region due to crack impediment and deflection at the intercrystalline organic interfaces (Fig. 7a, b; see Supplementary Fig. 19 for indentation curves), whereas similar loading on geological calcite induced significant radial cracking (Fig. 7a, inset). Cross-sectional TEM imaging of an indentation site close to an organic interface further confirms that the damage was effectively constrained by the organic interfaces even beneath the surface, and adjacent prisms were undamaged (Fig. 7c). Moreover, the characteristic nanoscale saw tooth-like structure at the mineral-organic interface, may lead to improved adhesion between the prisms and organic interfaces (Fig. 7d)^60^. Similar jagged interfaces with characteristic sizes of several micrometers were previously observed after etching, which was considered as the evidence of cyclic growth of calcitic prisms^16,61^. Together with the gradual upward change in defect orientation close to the prism boundaries, the *A. rigida* biogenic calcite is able to increase the density of microcracking and facilitate crack propagation upwards, thus avoiding deep fracture into the shell interior (Fig. 7e mechanism iii). With effective large-scale damage localizing effects provided by the intercrystalline interfaces, the individual calcite prisms often undergo extensive fracture into nano-/microscopic (*ca.* 200 nm) pieces (Fig. 7f), which further enhances energy dissipation by generating more fracture surfaces as well as promoting rotation and friction between fractured pieces^37^. Such simultaneous restriction in crack propagation via intercrystalline boundaries and extensive fracture within individual prisms can also be observed in macroscopic compression and indentation through synchrotron-based in situ measurements and post-deformation structural analysis (Supplementary Note 9 and Supplementary Figs. 20 and 21).

## Discussion

Nanoscopic intracrystalline defects are ubiquitous in a variety of biominerals from a wide range of taxa^11,62–65^, and the work presented here for *A. rigida* highlights the simultaneous intrinsic strengthening and toughening effects of this common structural motif. Our experiment- and modeling-based analysis indicates the spatially controlled morphology and distribution of intracrystalline defects guide and control the local fracture behavior of the *A. rigida* biogenic calcite. This deformation mechanism may underlie the primary structural origin for the observed “conchoidal” fracture of biogenic calcites, where the structural characteristics of intracrystalline defects are highly correlated to the biomineralization process in 3D^16,66,67^. The intrinsic mechanical properties of biogenic calcite revealed here can be combined with the extensive knowledge of the hierarchical organization in biological structural materials, which will enable a further in-depth understanding of the mechanical design principles in these materials^1,3–5^. As the majority of current efforts of bio-inspired structural materials have been directed to mimicking the assembly pattern and architectural organization of biological composites, the internal microstructure and hence the mechanical properties of individual building blocks, which are usually ceramic-based reinforcements, are often left unattended^3^. This work thus provides new insights into the development of damage-tolerant bio-inspired composites through the rational engineering of the internal microstructure of their ceramic phases.

## Methods

### Samples

Live *A. rigida* shells were purchased from Gulf Specimen Marine Laboratories, Inc. FL, USA. The specimens were frozen upon receiving and maintained under wet conditions prior to the experiments. Samples of single crystal geological calcite (origin, Mexico) were obtained from Pisces Trading Company, LLC.

### Electron microscopy

Samples were coated with ultra-thin carbon or Au to reduce charging effects prior to imaging with an FEI (Hillsboro, Oregon, USA)Helios Nanolab 660 Dual Beam scanning electron microscope (SEM) at an acceleration voltage of 5 kV and a working distance of *ca.* 4 mm. Energy dispersive spectroscopy (EDS) measurements were acquired from the same system equipped with an INCAEnergy EDS system at an acceleration voltage of 20 kV. Low-magnification SEM images shown in Fig. 7 were acquired with Tescan (Brno, Czech Republic) Vega GMU SEM with acceleration voltage of 20 kV. Cross-sectional transmission electron microscope (TEM) samples were prepared using ion beam milling. A detailed sample preparation procedure is as follows: (1) A platinum protective layer (*ca.* 0.5 μm) was first laid down on top of the desired region; (2) Another platinum protective layer (*ca.* 1.5 μm) was further deposited on top of the region where the TEM slab was to be milled out; (3) Two trenches, one on each side of the platinum protective stripe, were milled by FIB, leaving the specimen slab (thickness: *ca.* 1.5 μm); (4) The slab was then cut through by FIB and transferred to a copper TEM grid by an Omniprobe and welded securely with platinum deposition; (5) The lift-out lamellar specimen was sequentially thinned by FIB at 30, 16, 5, and 2 kV ion beam voltages. Final cleaning at 2 kV and 28 pA is important to obtain a clean surface and minimize damage. Imaging with standard bright-field, dark-field, and SAED techniques was carried out using a JEOL (Akishima, Japan) 2011 TEM operated at 120 kV.

In situ fracture experiments inside the SEM and fracture of TEM samples were conducted with a Helios Nanolab 600 Dual Beam scanning electron microscope equipped with an Omniprobe system. For in situ SEM imaging of fracture surface, a sample with fractured surfaces was first coated with ultra-thin carbon. After an area of interest was located, the Omniprobe was introduced and used to fracture off micron-sized portions of the sample, revealing fresh fracture surfaces. The newly fractured surface was then immediately imaged with SEM in immersion mode with acceleration voltage of 2 kV. For the fracture experiment of TEM samples, TEM imaging was first conducted on the samples before fracture. Within the Dual Beam microscope, the one side of the TEM sample was welded with the Omniprobe and pulled to fracture along the plane direction. The same area was finally imaged with a TEM instrument, JEOL 2011 at 120 kV.

### Atomic force microscopy

Tapping mode AFM (TMAFM) imaging in ambient conditions was carried out using a Veeco Multimode SPM IIIA (Santa Barbara, California, USA) equipped with an AS-130 “JV” scanner. TMAFM imaging was conducted with NANOSENSORS Si TMAFM cantilevers (PPP-NCHR-10). Typical scan speed was 1–5 μm/s; other parameters were optimized upon tuning.

### Nanoindentation

The *A. rigida* shells were first thoroughly cleaned with DI water to remove algae and sand particles on the surface, and then the prismatic layer was removed from the shell with a razor blade. The dried pieces of the prismatic layers were then cut into small square-shaped plates (*ca.* 5 mm), and embedded in a room temperature cured epoxy (Spurr low viscosity embedding kit, Polysciences Inc.). Samples with two different orientations were prepared, i.e., parallel and perpendicular to the *c*-axis (longitudinal direction) of prisms. The embedded samples were then polished on a  South Bay Technology (San Clemente, California, USA) Model 920 lapping machine with aluminum oxide pads stepwise (15 µm, 6 µm, 3 µm, and 1 µm), and finally with 50 nm silica colloidal solution on a microcloth. Load-controlled nanoindentation was performed on the polished sample surface by using Berkovich (trigonal pyramid, semi-angle = 65.3°) and conospherical (tip radius = *ca.* 1 µm, semi-angle = 30°) diamond probe tips on a Hysitron TriboIndenter (Minneapolis, Minnesota, USA). The piezoelectric transducer was first allowed to equilibrate for 105 s (the last 45 s with digital feedback) and another 40 s for calculating drift automatically prior to each indent. Typical load functions included loading (10 s), holding (20 s), and unloading (10 s). Maximum loads varied from 1 to 10 mN. The standard Oliver-Pharr (O-P) methodology was used to quantify material properties, i.e. indentation modulus (*E*_*O-P*_) and hardness (*H*_*O-P*_)^68^. The probe tip area function *A*(*h*_*c*_), which is the projected area of the indentation tip as a function of the contact depth *h*_*c*_, and frame compliance were calibrated prior to each set of experiments using a fused quartz sample. For the measurements of materials properties (*E*_*O-P*_ and *H*_*O-P*_) and size of fracture patterns, nanoindentation experiments were performed in the center of prisms with diameter greater than 30 µm to reduce edge effects. A number of indentation experiments were carried out close to intercrystalline organic interfaces to study the damage localization properties (Fig. 7c).

### Microindentation

Biogenic calcite (*A. rigida*) sample preparation for microindentation followed the same procedure as that used for nanoindentation. Micro-indentation was conducted on the polished transversal section of *A. rigida* shell (perpendicular to *c*-axis) by using a conospherical tip (tip radius = *ca.* 1 µm, semi-angle = *ca.* 30°, Micro Star Technologies, Huntsville, TX, USA) on a Nano Test Vantage platform 4 from Micro Materials (Wrexham, UK). To obtain the equivalent surface for micro-indentation, sample preparation for geological calcite required additional steps, including sample cutting to expose and confirm the {0001} surface before embedding and serial polishing. Typical load functions include loading (10 s), holding (10 s), and unloading (10 s). The maximum loads chosen were 10 and 50 mN, and thermal drifting was monitored when the load was unloaded to 10% of the maximum force for 30 s. To obtain direct comparison of fracture patterns, indentations were conducted within individual prisms of the *A. rigida* shell, and indents with cracks and fractured chips intercepting with crystalline boundaries were discarded. Finally, the fracture patterns were characterized by post-indentation SEM imaging using three parameters, *R*_*i*_, the radius of the inner indentation crater, *R*_*o*_, the radius of the entire fracture pattern by fitting it with the smallest circle, and *C*, the distance between the centers of the two fitted circles to characterize the eccentricity of damage.

### Macroscopic compression

Macroscopic compression experiments on mm-sized *A. rigida* and geological calcite samples were performed with an Instron (Norwood, Massachusetts, USA) 5984 universal testing machine. Isolated pieces of the prismatic layers of *A. rigida* shell were first polished to ensure that the top and bottom surfaces were flat and parallel. They were then cut into square-shaped pieces with sizes of *ca.* 3 mm, and the side surfaces were polished again before being dried in air. Geological calcite samples were prepared by cleaving along their {10$\bar 1$4} plane, and as such, the sidewalls of these samples were not perpendicular to the horizontal plane. The compression experiments were conducted using a displacement-controlled condition, with a typical displacement rate of 5 μm/s.

### Synchrotron in situ mechanical testing

Sample preparation for in situ tests followed the same procedure as that for macroscopic compression by removing the nacreous layer and organic contaminants and polishing. In situ tests were conducted using the micro-tomography instrument at beamline 2-BM of the Advanced Photon Source, Argonne National Laboratory, Chicago. The X-ray beam energy used was 27.4 keV with imaging resolution of 1.3 μm/pixel. A custom-built in situ holder with an alumina tip was designed to mount on the rotating stage with a linear actuator on top to add displacement-controlled loading (Supplementary Fig. 21a). Between loading steps, the stage was rotated 180° to obtain 1500 projections for reconstructing the original and deformed structure at each step. Tomographic reconstructions were performed using TomoPy^69^ and rendered with the  Thermo Fisher Scientific (Waltham, Massachusetts, USA) Avizo software package.

### Preparation of micro-pillars

The polished samples were coated with ultra-thin carbon to reduce charging effects. The micro-pillars were prepared within individual prisms using focused ion beam milling using a Helios 600 and a 660 Dual Beam Nanolab. A top-down annular milling procedure was used with an accelerating voltage of 30 kV and currents ranging from 2.5 nA to 28 pA. Prisms with cross sectional size greater than the diameter of the circular trenches, i.e. 30 μm, were selected for micro-pillar preparation. The depths of the circular trench surrounding the micro-pillars were greater than 2 µm so as to provide clearance for micro-compression testing. Cylindrical pillars were produced with diameters of 2–3 μm, aspect ratios of *ca.* 3, and a tapering angle of *ca.* 2.5°.

### Uniaxial compression of micro-pillars

Micro-compression experiments were conducted in ambient conditions using a Hysitron TriboIndenter (Minneapolis, Minnesota, USA). Uniaxial compression was performed using a flat-punch conical diamond tip (end diameter *D*_tip_ = 10 μm, cone half angle = 60°). The piezoelectric transducer was first allowed to equilibrate for 105 s (the last 45 s with digital feedback) and another 40 s for calculating drift automatically prior to each compression. Both depth and load controlled compression tests were performed with displacement and load rate of 10 nm/s and 0.4 mN/s, respectively.

### Data analysis for uniaxial micro-pillar compression

The as-measured force, *F*, and compression depth, *δ*, were converted to normalized engineering stress, *σ*_*n*_, and strain, *ε*_*n*_, by applying Sneddon’s formula, which accounts for the substrate compliance for a slightly tapered pillar^23^:

2 $$\varepsilon _n = \frac{\delta }{h}$$

3 $$\sigma _n = \left( {\frac{4}{{\pi D_1D_2}} + \frac{{1 - v^2}}{{D_2h}}} \right)F$$

where *h* is pillar height, *D*_1_ and *D*_2_ are the top and bottom diameter of micro-pillars, and the Poisson’s ratio, *ν*, is approximated as the average Poisson’s ratio of calcite (0.3)^70^. The elastic modulus, *E*, was extracted from the elastic portion of the stress-strain curves via a least-squares linear regression. Due to the slight nonlinearity, the 50–95% portions of the loading curves were used for the fitting. The yield strength, *σ*_*Y*_, and geometrical maximum stress, *σ*_max_, were estimated as

4 $$\sigma _Y = \frac{{4F_Y}}{{\pi D_1^2}}$$

and

5 $$\sigma _{\max } = \frac{{4F_{\max }}}{{\pi D_1^2}}$$

where *F*_*Y*_ is the onset load of nonlinear portion, and *F*_max_ is the maximum load. A Weibull distribution model^57^ was used to analyze the materials’ strength, *σ*_*Y*_, by fitting the failure percentage, *f*, as

6 $$f = 1 - e^{ - (\frac{{\sigma _Y}}{{\sigma _{Y0}}})m}$$

where *σ*_*Y*0_ and *m* (Weibull constant) are fitting parameters^71^.

### Molecular dynamics simulations

Molecular dynamics (MD) simulations were performed using the large-scale atomic/molecular massively parallel simulator (LAMMPS)^72^. The Ovito visualization tool was used for post processing the results of MD simulations^73^. The calcite pillars were shaped by cutting a cylinder with diameter *d* = 200 Å out of a periodic cube with the initial size of 450 × 450 × 450 Å. The pillar’s axis was set along the *c*-axis of the calcite crystal. The total charge of the system was kept neutral in the pillar by cutting the cylinder in a way such that each cross section contained an equal number of calcium and carbonate groups. The pillar with no defects, simulating pure geological calcite, contained a total of 1,102,400 atoms. In the sample with ellipsoid defects (simplified as a void), representing biogenic calcite with nano-inclusions, atoms in an ellipsoid containing 218 CaCO_3_ units were deleted inside the cylinder. Interatomic forces in calcite were described by a rigid ion version of the interatomic potential reported by Pavese et al.^74,75^, which has been shown to be capable of accuratelypredicting various mechanical properties of calcite^76^. The simulation time was selected as 1 fs, and thermal equilibrium was applied to the system using time integration on Nose-Hoover style non-Hamiltonian equations of motion in canonical (NVT) ensembles to set the temperature at *T* = 10 K for 100 ps. Tracking the pressure along the periodic direction showed that the system was equilibrated and the stress along the axis was zeroed after 100 ps. The pillar was then loaded in compression up to 30% by continuously changing the box volume along the periodic direction with a constant strain rate of 3 × 10^8^ s^−1^. During loading the NVT with Nose-Hoover thermostat was applied to maintain the temperature constant.

## Supplementary information

Supplementary Information

## Data Availability

The main data supporting the findings of this study are available within the Article and Supplementary Information files. Additional data are available from the corresponding authors on reasonable request.
