# Supplementary material for: Strategies for simultaneous strengthening and toughening via nanoscopic intracrystalline defects in a biogenic ceramic
Source: Nat Commun. 2020 Nov 10;11:5678. doi: 10.1038/s41467-020-19416-2 (PMC7655841; doi:10.1038/s41467-020-19416-2)
Supplement: Supplementary file 1 — Supplementary Information [file 41467_2020_19416_MOESM1_ESM.pdf]

Supplementary information
for

Biological strategies for simultaneous strengthening and toughening via nanoscopic intracrystalline defects in a biogenic ceramic

Zhifei Deng[1], Hongshun Chen[1], Ting Yang[1], Zian Jia[1], James C. Weaver[2], Pavel D. Shevchenko[3], Francesco De Carlo[3], Reza Mirzaeifar[1], and Ling Li[1,*]

[1]Department of Mechanical Engineering, Virginia Polytechnic Institute and State University, 635 Prices Fork Road, Blacksburg, VA 24061 USA.
[2]Wyss Institute for Biologically Inspired Engineering, Harvard University, 60 Oxford Street, Cambridge, MA 02138, USA.
3 Advanced Photon Source, Argonne National Laboratory, 9700 S Cass Ave, Lemont, IL 60439, USA

*To whom correspondence should be addressed. E-mail: lingl@vt.edu.

Table of Contents

1. Supplementary notes 1-9
    1. Intracrystalline inclusions: geometrical and orientational quantifications
    2. Spacing between intracrystalline inclusions
    3. Calculation of Schmid factor
    4. Investigation of the slip systems in geological calcite
    5. Strengthening effect from the intracrystalline inclusions
    6. Stress distribution around intracrystalline defect-like inclusions
    7. Quasi-brittle fracture of biogenic calcite
    8. Investigation of the fracture behavior via FE/DE simulations
    9. Intercrystalline organic interfaces: confining damage and promoting fracture within prisms
2. Supplementary figures 1-24
3. Supplementary references 1-18

Supplementary note 1

Intracrystalline inclusions: geometrical and orientational quantifications

Geometrical quantifications
Bright-field TEM images (typical magnification, ~50k) of the biogenic calcite taken in two orientations were used for the quantification (**Supplementary Fig. 3a,d**). Individual defect-like inclusions were first contoured manually (**Supplementary Fig. 3b,e**), and the edge profiles of all contoured inclusions were overlaid by aligning their centers (**Supplementary Fig. 3g**). An averaged edge profile was obtained by averaging the edge locations at each given polar angle (every 5 degrees, **Supplementary Fig. 3h**). Finally the averaged edge profiles were fitted with an ellipse with principle axes of *a* and *b* along the transversal and vertical directions, respectively (**Supplementary Fig. 3i,j**). Note that the fitted results based on manual segmentation represents the upper bound as small-sized inclusions were ignored in manual segmentation.

Orientational quantifications
Based on stitched bright-field TEM images taken from the longitudinal cross section near the intercrystalline organic boundary (**Supplementary Fig. 4a**), the location-dependent orientation of intracrystalline organic defects was analyzed through the following procedure:

First, segmentation of inclusions was done using an open-source software (ilastik, version 1.3.2) integrated with machine learning based-training (**Supplementary Fig. 4b**). Next, the nano-defects were treated as particles and analyzed using another open-source software (Extended Particle Analyzer, BioVoxxel plugin, ImageJ), in which each separated particle was registered and fitted using an ellipse (**Supplementary Fig. 4c,d**) with shape parameters exported, including inclination angles, coordinates of the centroid, long and short axes, etc. Note that we consider the inclination angles range from -90° to 90° (**Supplementary Fig. 4d**). Then, to reduce the noise level, all the particles with aspect ratio less than 2.026 (= 5.47/2.70, the aspect ratio of manual shape analysis results in **Supplementary Fig. 3j**) were filtered.

The high density of inclusions near the prism boundary causes overlapping in segmented results (**Supplementary Fig. S4f**), therefore, the defects within 100 nm from prism boundary was removed for fitting analysis. The orientation of the identified intracrystalline defects was fitted with an exponential function as following,

$$\theta(x) = 50.23e^{1.018\times10^{-3}x} \quad (1),$$

where θ is the inclination angle with respect to transversal direction, and x (in unit of nm) is the negative distance from the prism boundary ($x = 0$). The fitted orientation curve shows close match to the original TEM images at different locations (**Supplementary Fig. 4e**). With this fitting result, the orientation of intracrystalline defects close to the prism boundary is ca. 50°. The defects become approximately horizontal (~2°-3°) when they are approximately 3 μm away from the prism boundary.

Supplementary note 2

Spacing between intracrystalline inclusions

Volume percentage of organic inclusions

Nudelman *et al*. conducted a detailed compositional analysis of the organic matrix in the prismatic layer of *A. rigida* through the direct extraction method[1]. Based on this analysis, it was found that the total organic materials in the prismatic layer (both inter-prismatic and intra-prismatic organics) is 5.645 *wt*%, where the intra-prismatic (or intra-crystalline) organics is 0.365 *wt*%, including 0.085 *wt*% and 0.28 *wt*% of soluble and insoluble organic materials, respectively. Based on this analysis, the weight percentage of intra-crystalline organics within the mineral matrix is 0.385 *wt*% as calculated by using following relationship

$$wt\% = \frac{wt_{organics,intra}\%}{wt_{organics,intra}\% + wt_{calcite}\%} \quad (2).$$

Therefore, the corresponding volume percentage can be calculated by using the following relationship

$$\frac{vol\%\rho_{organics}}{vol\%\rho_{organics} + (1 - vol\%)\rho_{calcite}} = wt\% \quad (3).$$

Assuming the densities of the organics and calcite are 1 and 2.71 g/cm^3, respectively, the volume percentage of the intra-crystalline organic materials is 1.03 *vol*%.

Estimation of spacing between individual inclusions

From the measured geometry and size of inclusions, the averaged shape of the inclusions can be regarded as a squashed sphere with long and short axes of 5.47 and 2.70 nm, respectively. This corresponds to a sphere with a radius of 4.32 nm for the same volume. Assuming the defects are dispersed in a cubic lattice, the average spacing between the adjacent inclusions can be estimated from

$$\text{total intracrystalline organic inclusions } vol\% = \frac{\text{volume of one inclusion}}{\text{inclusion spacing}^3} \quad (4),$$

which gives the estimated spacing of ~32 nm.

Note that this estimation should be the upper bound, since the estimation of averaged volumes of individual defects is overestimated since smaller inclusions are ignored in manual segmentation **(Supplementary note 1)**. The result is in general agreement with TEM results, where the spacing is typically in the range of ~20-40 nm.

Supplementary note 3

Calculation of Schmid factor

Throughout this work, we use Miller-Bravais crystallographic indices referred to the hexagonal unit cell of calcite with lattice parameters a = 4.99 Å, and c = 17.06 Å. We use the notation x$(hkil)[uvtw]^{\pm}$ and x$\{hkil\}\langle uvtw\rangle^{\pm}$ to specify individual slip systems and families of systems, respectively. Superscripted signs were used to indicate slip sense, following previous conventions[2,3].

Main plastic deformation modes in calcite including both slip and twinning are summarized in **Supplementary Table 1**. The known Burger's vector, B, for different slip systems are included[3,4]. Under the uniaxial compression along the c-axis, the angle between the loading direction and slip direction, λ, and the angle between the loading direction and the normal direction of the slip plane, ϕ, are calculated. The corresponding Schmid factor is given through the following Schmid law

$$m = \cos\phi \cos\lambda \tag{5}$$

Based on these calculations, the Schmid factor for the r-slip $\{10\bar{1}4\}\langle\bar{2}021\rangle^{\pm}$ has the highest value (m = 0.49996).

Deformation mode	System	Number of systems	B	λ	ϕ	Schmid factor
**Twinning**	$e\{\bar{1}018\}\langle 40\bar{4}1\rangle^{+}$	3	-	63.74	26.26	0.39683
	$r\{10\bar{1}4\}\langle\bar{2}021\rangle^{\pm}$	3	$1/3\langle\bar{2}021\rangle$, 0.77 nm	45.38	44.62	0.49996
	$f\{\bar{1}012\}\langle 2\bar{2}01\rangle^{\pm}$	3	$1/3\langle 10\bar{1}\bar{1}\rangle$, 0.81 nm	45.38	63.13	0.31746
**Slip**	$f\{\bar{1}012\}\langle 0\bar{2}2\bar{1}\rangle^{\pm}$	3	-	45.38	63.13	0.31746
	$f\{\bar{1}012\}\langle\bar{1}01\bar{1}\rangle^{\pm}$	3	-	26.87	63.13	0.40315
	$c\langle a\rangle$	3	$1/3\langle 2\bar{1}\bar{1}0\rangle$, 0.49 nm	90	0	0

Supplementary Table 1. Twinning and slip systems in calcite. B is the Burger's vector. λ is the angle between the loading direction (c-axis) and slip direction. ϕ is the angle between the loading direction (c-axis) and the normal direction of the slip plane.

Supplementary note 4

Investigation of the slip systems in geological calcite

Under significant plastic deformation, the micro-pillars of geological calcite, in some cases, underwent plastic flow within a single *r*-slip system, as shown in **Supplementary Fig. 8**. In this case, the top portion of the micro-pillar was shifted laterally during the test, while the bulk shape of the pillar maintained integrity (**Supplementary Fig. 8b,e**). Notice that there is no significant strain hardening during the plastic flow regime in this case (**Supplementary Fig. 8d**).

In the plastic flow regime, micro-pillars of geological calcite may also undergo significant slip motions in two or three equivalent *r*-slip systems (**Supplementary Figs. 9,10**, respectively). In this case, the micro-pillars did not undergo significant lateral shift due to the interaction among multiple slip systems. Also, with the three-fold symmetry of the *r* systems, the slip lines appear different morphologies when viewed from different angles (**Supplementary Fig. 9d,e**). This also allows a direct comparison between experimental observations and 3D geometrical models. In the geological calcite pillars with three-equivalent slip systems, since the *c*-axis is aligned with the pillar axis, the three-fold symmetry can be viewed from the top-viewed SEM image of the tested micro-pillar (**Supplementary Fig. 10d-f**). The three lobes extending out are resulted from slips, where adjacent lobes are 120 degrees apart. The slip lines can be clearly observed in each *r*-slip system, which exhibit the same morphology as the 3D geometric model (**Supplementary Fig. 10g-j**).

If the compression was immediately stopped and unloaded after yielding, the geological calcite exhibited permanent deformation in the top portion of the micro-pillars (**Fig. 4f**). **Supplementary Fig. 11a,b** show a micro-pillar of geological calcite before testing and immediately stopped after yielding, respectively. The corresponding stress-strain curve is shown in **Supplementary Fig. 11c**. The TEM thin section prepared from the deformed pillar confirmed that the permanent deformation was concentrated at the top portion of the micro-pillar (**Supplementary Fig. 11d,e**). No significant micro-cracking was observed within the bulk micro-pillar.

Supplementary note 5

Strengthening effects from the intracrystalline inclusions

To quantify the strengthening effect of the intracrystalline inclusions, the relationship between the increase of hardness and yield strength in biogenic calcite compared with geological calcite is analyzed. Previous studies indicate that for materials with $\sigma_Y/E < 0.02$, hardness H approaches 2.8 σ_Y[5]. Following the analysis approach developed by Kim *et al.* for biomimetic calcite crystals, we can relate the increase in hardness due to the presence of defects with the increase in strength through the following relationship[6]

$$H_{inclusion} - H_{pure} \approx 2.8(\sigma_{Y,inclusion} - \sigma_{Y,pure}) = 2.8\Delta\sigma_Y \quad (6),$$

where $H_{\mathrm{inclusion}}$ and H_{pure} represent the hardness of calcite with and without inclusions, respectively, and $\sigma_{Y,\mathrm{inclusion}}$ and $\sigma_{Y,\mathrm{pure}}$ represent the theoretical strength of calcite with and without inclusions, respectively.

This equation can be rewritten as

$$H_{inclusion} = H_{pure} + C\Delta\sigma_Y \quad (7),$$

where $C = 2.8$ is a constant.

Based on indentation measurements, the hardness values of biogenic and geological calcite are $H = 2.83 \pm 0.19$ GPa and $H_0 = 1.81 \pm 0.11$ GPa, respectively. The indentation results are smaller than published literature, which could result from azimuthal variations and the sharpness of the new Berkovich tip only in recent use, but the difference of indentation hardness measured for *A. rigida* and geological calcite ΔH matches well with previous reported results[7]. Therefore, with the presence of intracrystalline defects, the increase of hardness ($\Delta H = H - H_0 = 1.02$ GPa) should theoretically correspond to an increase of yield strength of $\Delta\sigma_{Y,t} \approx 0.36$ GPa, resulting a hypothetical yield strength of 0.96+0.36 GPa = 1.32 GPa, This value is higher than the measured results $\sigma_Y = 1.16 \pm 0.22$ GPa.

Supplementary note 6
Stress distribution around intracrystalline defect-like inclusions

Here we consider the local stress distribution around individual intracrystalline defects to understand their influence in crack initiation and propagation. The discussion is based on the classical Gordon-Cook principle[8].

First, we consider the stress intensity factors associated with individual inclusions in two orientations, K_l and K_s, along the long and short axes, respectively. Assuming a far-field stress field, σ, is present, cracks propagate along the long and short axes of the inclusions, respectively, as shown in **Supplementary Fig. 22**.

According to *Inglis* (1913)[9], the stress intensity factor for an elliptical defect is given by

$$K = 1 + 2\frac{a}{b} \qquad (8),$$

where a and b are semi-major and semi-minor axes, respectively.

The measured geometry and size of intracrystalline defects indicate (**Supplementary Fig. 3g-j**) that $a \approx 5.5$ nm, and $b \approx 2.7$ nm. Therefore, $K_l = 5.07$ and $K_s = 1.98$. The tendency for crack initiation along the defects' long axes is approximately 2.6 times than that along the short axes.

Next, we consider the tendency of crack initiation at the horizontal tip of intracrystalline inclusions due to the presence of the primary vertical primary cracks, as shown in **Supplementary Fig. 23**. Under a far-field stress field σ_0, the horizontal local stress σ_h opening the main crack is amplified, and the maximum of this stress occurs at the crack tip

$$\sigma_{h,\max} = (1 + 2\sqrt{l/r})\sigma_0 \qquad (9),$$

where l is half length of the primary crack, and r is tip radius of the primary crack.

In addition, the primary crack also generates a tensile stress field in the vertical direction (σ_v), and the maximum of this stress is at a distance roughly equal to one crack tip radius, and its values is slightly less than one-fifth of the maximum value of σ_h[8]

$$\sigma_{v,\max} = \frac{1}{5}(1 + 2\sqrt{l/r})\sigma_0 \qquad (10).$$

Considering an intracrystalline inclusion located at a distance r away from the primary crack, which is opened up under this local vertical stress, the final maximum resultant stress around the elliptical defect in the vertical direction is given by

$$\sigma_{v,inclusion} = \frac{1}{5}(1 + 2\sqrt{l/r})(1 + 2\frac{a}{b})\sigma_0 \approx (1 + 2\sqrt{l/r})\sigma_0 = \sigma_{h,\max} \qquad (11).$$

Therefore, we notice that the stress intensity level for the intracrystalline inclusions in the vertical direction approximates to the stress intensity level of the primary vertical crack. In this case, horizontal crack can be produced at the tip of the inclusions. Moreover, once the primary crack propagates to the intracrystalline defect-like inclusions, the curvature of the primary crack front is reduced due to the anisotropic geometry of inclusions, leading to reduced stress intensity along the vertical direction. This

will further promote the crack deflection into the horizontal directions, that is, the transversal planes in prisms.

The above analysis is based on the interaction of a primary vertical crack and a single elliptical defect. Presence of multiple defects in the transversal direction with similar anisotropic geometries will further enhance such effect, leading to even greater tendency of crack deflection into the transversal direction. Moreover, the above discussion is based on the average geometry of inclusions that have ideal ellipse geometries. Faceted boundaries with sharper corners, as shown in **Supplementary Fig. 17**, will further enhance the stress intensity levels in the vertical direction, which also contributes to the greater tendency of crack deflection into the transversal direction.

Supplementary note 7

Quasi-brittle fracture of biogenic calcite

In this note, we analyze and discuss the effects of the orientation, size, and spacing of the defects within the biogenic calcite on its strength, following the models originally developed by *Germanovich* and *Dyskin* for quasi-brittle materials with pre-existing defects[10].

We first consider a simple scenario with non-interacting defects, where the intracrystalline inclusions are treated as narrow pre-existing cracks situated far from each other within the calcite matrix (**Supplementary Fig. 24a**); in this case the secondary cracks can grow significantly with little or no interaction. The crack-opening forces (which opens the secondary cracks at the tips of the pre-existing crack) originate from the sliding of the opposite lips, as shown in **Supplementary Fig. 24a**. It is assumed that the secondary cracks grow in Mode I due to tensile stress concentrations at the inclusion tips, therefore, only forces that produce normal opening of the cracks are taken into account[10].

With an inclination angle of α (= 90°-θ) with respect to the compression direction and 2a as the characteristic length of the defect, the total normal force acting on the defect is

$$F_N = 2a\sigma \sin^2 \alpha \qquad (12),$$

where σ is applied compressive stress along the growth direction, and θ is the inclination angle of defects regarding the transversal direction, as shown in **Supplementary Fig. 24a**. The resultant Mode-I line force (parallel to the long axis of the defect, which opens the secondary cracks) is given by

$$F = 2a\sigma\beta(\alpha) \qquad (13),$$

where

$$\beta(\alpha) = (\sin\alpha)^2 \cos\alpha(1 - \tan\alpha \tan\mu) \qquad (14),$$

where μ is the friction angle between inclusions and the mineral matrix.

By using the Mode-I LEFM criteria for crack growth, the length of secondary cracks can be expressed as[53]

$$l = \frac{1}{\pi}\left(\frac{2\beta(\alpha)a}{K_{Ic}}\right)^2 \sigma^2 \qquad (15).$$

Assuming the composite crystal fails through fracture when secondary cracks start to coalesce (*i.e.*, $l = L$, where L represents the inclusion spacing. **Supplementary Fig. 24b**), the yield strength of the biogenic calcite can be estimated as following

$$\sigma_{y(\text{non-interacting})} = \pi^{1/2} L^{1/2} \left(\frac{K_{Ic}}{2\beta(\alpha)a}\right) \qquad (16).$$

Since the biogenic calcite crystal contains many pre-existing intracrystalline inclusions, the interaction between those defects needs to be considered. The following model, developed by *Germanovich* and *Dyskin* for prediction of compressive strength of brittle materials with distributed pre-existing cracks, can be used for predicting the effective yield strength[10]

$$\sigma_{y(\text{interacting})} = \left(\frac{2\pi}{11N}\right)^{1/4}\left(\frac{K_{Ic}}{2\beta(\alpha)a}\right) \quad (17),$$

where N is the average number of cracks per unit area. Here if we assume the intracrystalline defects are dispersed in a cubic lattice (**Supplementary note 2**), the relationship between N and L is given by

$$N = \frac{1}{L^2} \quad (18).$$

Therefore, we have

$$\sigma_{y(\text{interacting})} = \left(\frac{2\pi}{11}\right)^{1/4} L^{1/2}\left(\frac{K_{Ic}}{2\beta(\alpha)a}\right) \quad (19),$$

and

$$\sigma_{y(\text{interacting})} < \sigma_{y(\text{non-interacting})} \quad (20).$$

Here we note that the variation in the distribution and morphology of intracrystalline inclusions leads to the variations in the local strength of the mineral-inclusion composite. More specifically, at the boundaries of prisms, the defects are orientated with much higher inclination angles (*ca*. 40-50° based on TEM measurements, **Supplementary Fig. 4**), we would expect that the cracks are more easily formed in these regions. Two direct effects result from such a design: 1) this leads to easy fracture close to intercrystalline organic interfaces, which tends to produce a large number of fracture pieces with smaller sizes. This contributes to the enhanced energy dissipation and avoidance of large stress concentrations by distributing to many cracks. 2) Cracks, easily produced near the crystal boundary, further channel to the central regions of prisms in the transversal direction, forming extensive crack network for enhanced energy dissipation.

Secondly, along the vertical direction, the density variation of inclusions directly leads to the local micro-fracture in the high-density regions first (**Fig. 5e inset and Supplementary Fig. 14**). In addition, the transversal alignment of the high-density regions also ensures that the cracks, once initiated and coalesced, tends to propagate along the transversal direction, minimizing vertical damage to the entire shell.

Here we calculated $\sigma_{y(\text{interacting})}$ as a function of defect spacing, L, and inclination angle with respect of transversal direction $\theta = 90°-\alpha$, by using the following experimental results and estimated relations:

$\sigma_{Y,c} = 0.96$ GPa, compressive strength of calcite obtained from micro-pillar compression results.

$\sigma_{Y,t} \approx \frac{1}{10}\sigma_{Y,c}$, tensile strength estimation approximates to 10% of its compressive strength for brittle materials including ceramics[11,12].

$K_{Ic} = F\sigma_{Y,t}\sqrt{\pi a}$, fracture toughness of biogenic calcite based on classic fracture mechanics (Griffith's law), where F is a dimensionless parameter or function that depends on specimen and crack geometry. Considering a separated inclusion inside a prism (defect size a = 5.5 nm, prism diameter d = 20-50 μm) in our case, F = 1[13].

Thus, the equation for estimating strength considering the interaction between inclusions can be modified as

$$\sigma_{y(\text{interacting})} = \left(\frac{2\pi}{11}\right)^{1/4} L^{1/2} \left(\frac{\sigma_{Y,c}\sqrt{\pi a}}{20\beta(\theta)a}\right) \quad (21),$$

where $\beta(\theta) = (\cos\theta)^2 \sin\theta(1-\cot\theta\tan\mu)$, and $\tan\mu = 0.05$ is the frictional coefficient obetween organics and mineral matrix, defined as the ratio between the shear strength of organics (~50 MPa, estimated from the shear strength of organic interfaces in nacre[14,15] over the strength of calcite matrix.

This analysis allows us to estimate the strength of biogenic calcite in relation to the defect spacing and inclination angles (shown in **Fig. 6f**).

Supplementary note 8

Investigation of the fracture behavior via FE/DE simulations

In this note, we perform fracture simulations of biogenic calcite with different inclination angles to directly compare the fracture patterns.

Based on the geometrical quantification of intercrystalline inclusions in *A. rigida* prisms (**supplementary note 1**), three 2D models were cropped from the manual segmentation results of inclusions on the longitudinal cross section of the prism (**Supplementary Fig. 3f**) by rotating the pattern with different angles (0°, 30° and 50°) with respect to the center (**Supplementary Fig. 18a**). Frequency histograms show that the orientations of defects within each model approximately correspond to cropping rotation (**Supplementary Fig. 18b**). Additional 2D thin platens were added to the top and bottom of the models (**Supplementary Fig. 18d**); a constant velocity of 0.5μm/μs was applied to the top plate, and load-displacement results of the top platen can be accumulated during the fracture simulations, which were later converted to stress-strain curves for different models, respectively.

Fracture simulations were carried out using Irazu (Geomechanica Inc.), a package to perform fracture simulations using a combined finite-discrete element method (FDEM)[16], where the defect-like inclusions were simulated as voids or pre-existing cracks. All the 2D-models have the same dimension (252 nm × 252 nm) divided into ~150,000 triangular elements. Fracture is allowed to initiate at and propagates along the cohesive element at the interface between adjacent finite elements. Tensile strength (σ_t) and shear strength (f_s) control the initiation of fracture, while mode I and mode II fracture energy dictate the fracture behavior[17].

$$f_s = c + \sigma_n \tan\varphi \qquad (22),$$

where c is the cohesive strength, φ is the fractional angle and σ_n is the normal stress applied on the interface of elements. After cracking, the interaction between fragmentations is also considered in the simulations via a robust contact algorithm. Input parameters include Mode I fracture energy (0.32 J/m^2 of calcite), Poisson's ratio (0.3), tensile strength (450 MPa, based on bending experimental results of individual prisms from *A*. rigida), and fine-tuned parameters (Young's modulus 90 GPa, cohesion 750 MPa, and Mode II fracture energy 0.2 J/m^2) to ensure comparable mechanical properties of 0°-model with micro-pillar compression experiments.

The simulations were stopped and the models were considered completely fractured when a large volume of catastrophic failure occurred, evidenced as the sharp load drop shown in the stress-strain curves (**Supplementary Fig. 18c**). And mechanical properties for each model are extracted in **Supplementary Table 2**, as well as the defect area fraction. Note that the models from TEM segment should include more defects than actual structure because of overlapping effect of inclusions within TEM thin slice.

Typical fracture evaluation for each model at different strain levels is shown in **Supplementary Fig. 18d** and **Supplementary Videos 1-3**; despite the relatively high compressive velocity used, the prevention of stress waves in the models was ensured. It can be concluded that cracks first initiate at regions with high density of defects in all cases. Close-up images at crack-initiation sites also demonstrate: 1) wing cracks form at the sharp end (long axis) of inclusions along the vertical direction (**Supplementary Fig. 18e**); 2) cracks initiate by coalescing the sharp ends of adjacent defects, especially

with short spacing (**Supplementary Fig. 18f-i**); 3) for models with higher inclination angles, the spots with higher tensile stress are easier to form band-like region, with crack channeling multiple pre-existing defects.

	Defect area fraction	Young's modulus (GPa)	Fracture Strength (GPa)	Failure Strain
0°-model	10.11 %	34.478	1.200	0.070
30°-model	13.05 %	36.331	0.778	0.049
50°-model	14.24 %	39.467	1.066	0.050

Supplementary Table 2. Fracture simulation results of 2D models with defects of three different inclination angles, including 0°, 30°, and 50°.

Supplementary note 9

Intercrystalline organic interfaces: confining damage and promoting fracture within prisms

In this note, we demonstrate the deformation behavior of *A. rigida* prismatic layer at a higher level of structural hierarchy by presenting experimental results from macroscopic compression, and synchrotron-based *in-situ* indentation.

Macroscopic compression
Sample preparation procedure and testing conditions are included in **Methods**. From direct comparison of the compression curves in **Supplementary Fig. 20a**, it shows that the calcitic prismatic layer in *A. rigida* exhibits a much higher strength than geological calcite (328.44 ± 65.72 vs. 63.30 ± 29.85 MPa), and continues to sustain load beyond yield points, whereas the geological calcite fails catastrophically. SEM images of tested samples show that geological calcite develops large cracks following the cleavage planes of calcite (**Supplementary Fig. 20b,d**), while the biogenic calcite still retains the general integrity due to the presence of organic interfaces despite fracturing into nano-/microscopic (~200 nm) pieces (**Supplementary Fig. 20c,e**).

Synchrotron-based in-situ indentation
In-situ indentation test with an alumina tip was conducted to create concentrated loading configuration (**Supplementary Fig. 21a**). Between loading steps, the stage was rotated 180° to scan the deformed structure at different stages. With a resolution of 1.3 μm/pixel, the intercrystalline organic interfaces can be cleared captured, as well as the role in constraining the damage during deformation (**Supplementary Fig. 21b**).

A general observation shows that initial contact with concentrated loading led to localized damage of the prisms surrounding the tip, where the interface openings (intercrystalline organics) prevent extensive damage propagation (**Supplementary Fig. 21c,e**). Under severe deformation, the indentation loading caused extensive deformation and transecting cracks across the entire sample (**Supplementary Fig. 21d,f**). However, the failed prisms were then fractured into micrograins for extensive energy dissipation, and cracks deflected along the horizontal direction were observed which contribute to structural integrity by avoiding crack perforation across the shell thickness.

Supplementary Figures

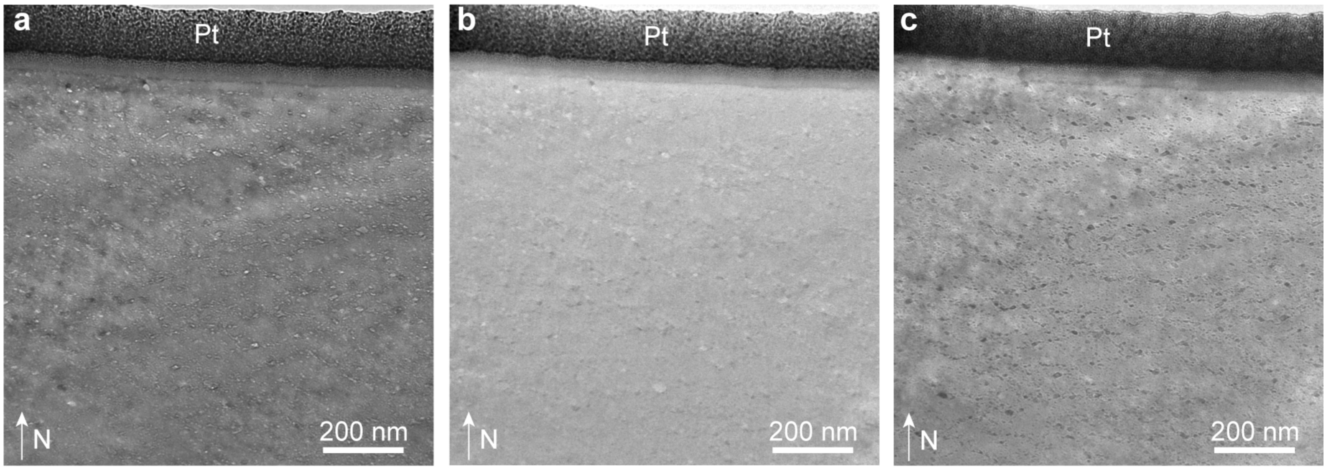

Supplementary Fig. 1. Observations of intracrystalline inclusions in biogenic calcite based on bright-field TEM imaging with different focus conditions: (**a**) underfocused; (**b**) nearly in-focus; (**c**) overfocused. When the beam is underfocused, the inclusions appear white with dark fringes; when the beam is overfocused, they appear dark with white fringes. When the specimen is nearly in focus, there is minimum contrast between the inclusions and the mineral matrix. This effect of contrast results from the difference in electron density between the organic inclusions and the mineral matrix, which causes a phase shift in the transmitted electron plane waves. This phase difference causes Frensnel fringes to develop along the edges of the intracrystalline defects[18].

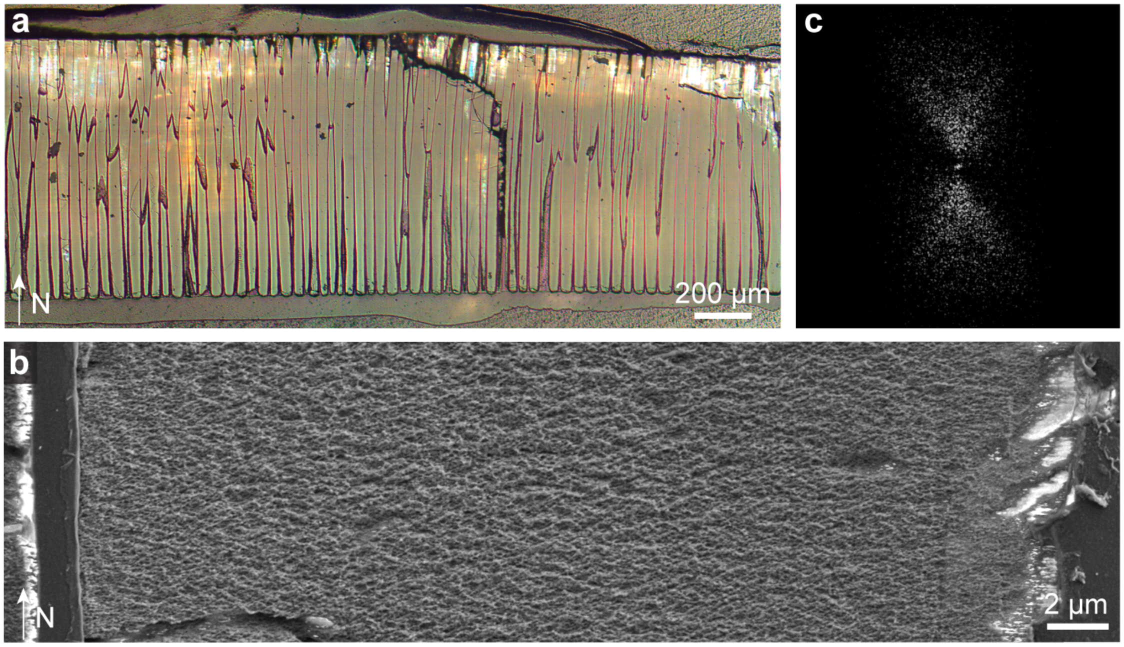

Supplementary Fig. 2. Visualization of the anisotropic distribution of intracrystalline organic materials from the polished and then etched surface. (**a**) Optical image of a prismatic layer polished along the normal direction before etching. (**b**) SEM image of the crystal surface after etching with HCl (1M) for 1 min. The sample was coated with ultra-thin carbon to reduce the charging effect. (**c**) Corresponding Fast Fourier Transform (FFT) pattern based on (**b**), showing the anisotropic nature of the etched surface.

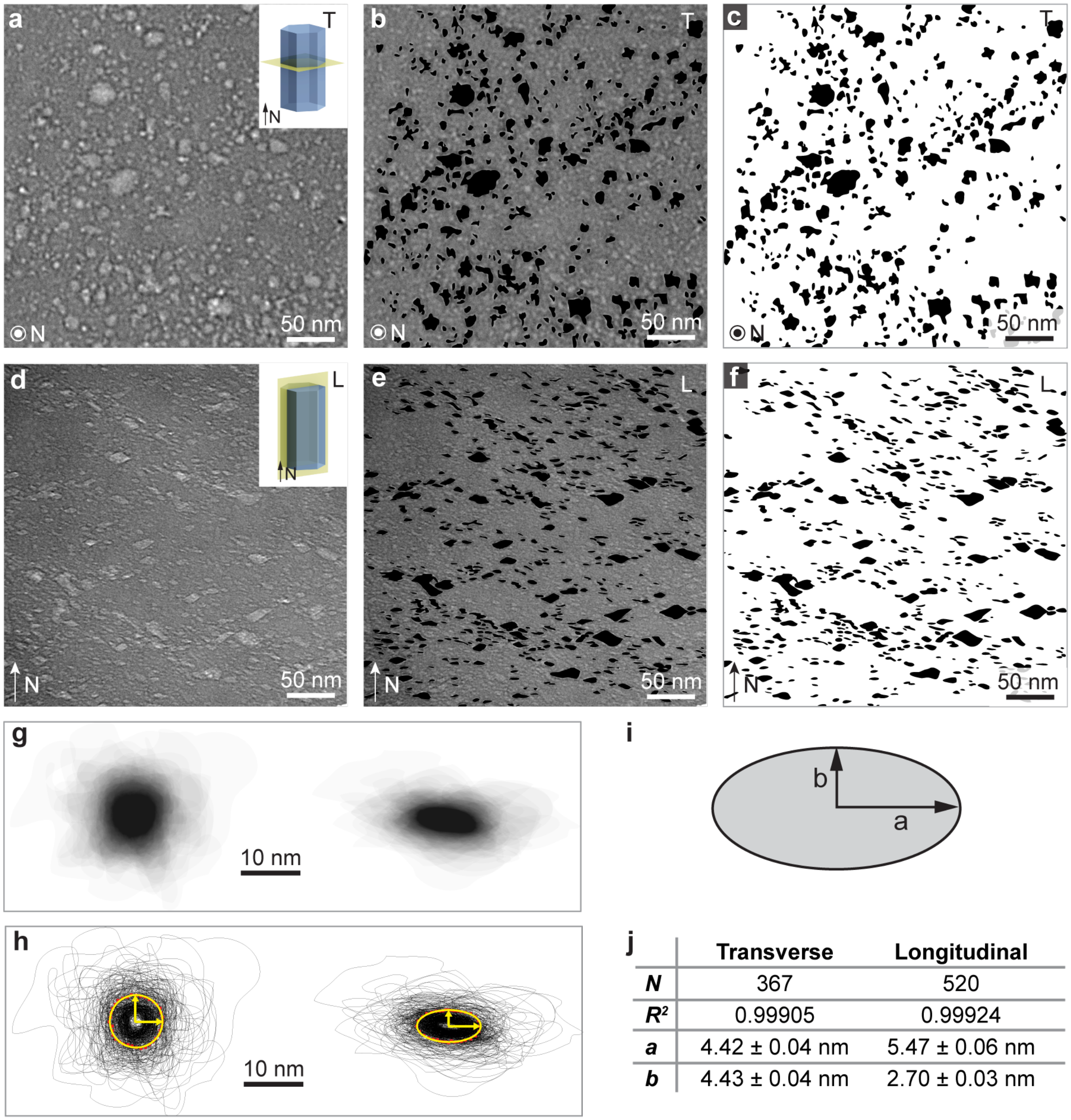

	Transverse	Longitudinal
N	367	520
R^2	0.99905	0.99924
a	4.42 ± 0.04 nm	5.47 ± 0.06 nm
b	4.43 ± 0.04 nm	2.70 ± 0.03 nm

Supplementary Fig. 3. Geometric quantification of the inclusions based on TEM imaging. (**a**,**d**) Representative bright-field underfocused TEM images in two orientations. (**b**,**e**) TEM images with inclusions overlaid by manual segmentation, which are represented by black particles. (**c**,**f**) Corresponding images with segmented inclusions. (**g**) Images with all the segmented inclusions overlaid on top of each other for two orientations. Here the centers of all the segmented inclusions were used for alignment. Each inclusion was set as a semi-transparent image, and the overlapped regions increase in the blackness that represents qualitatively the average geometry of all the defects. (**h**) Contours of all the segmented inclusions overlaid on top of each other for two orientations. The average geometry of defects can be estimated by calculating the average radius at a given polar angle, shown by the red dotted points in **Fig. 2g,h**. Lastly, the average geometries were fitted with an ellipse, shown by the yellow profile. (**i**) The illustration of ellipse with two principle axes, *a* and *b*, used for profile fitting. (**j**) Statistics of the ellipse fitting results.

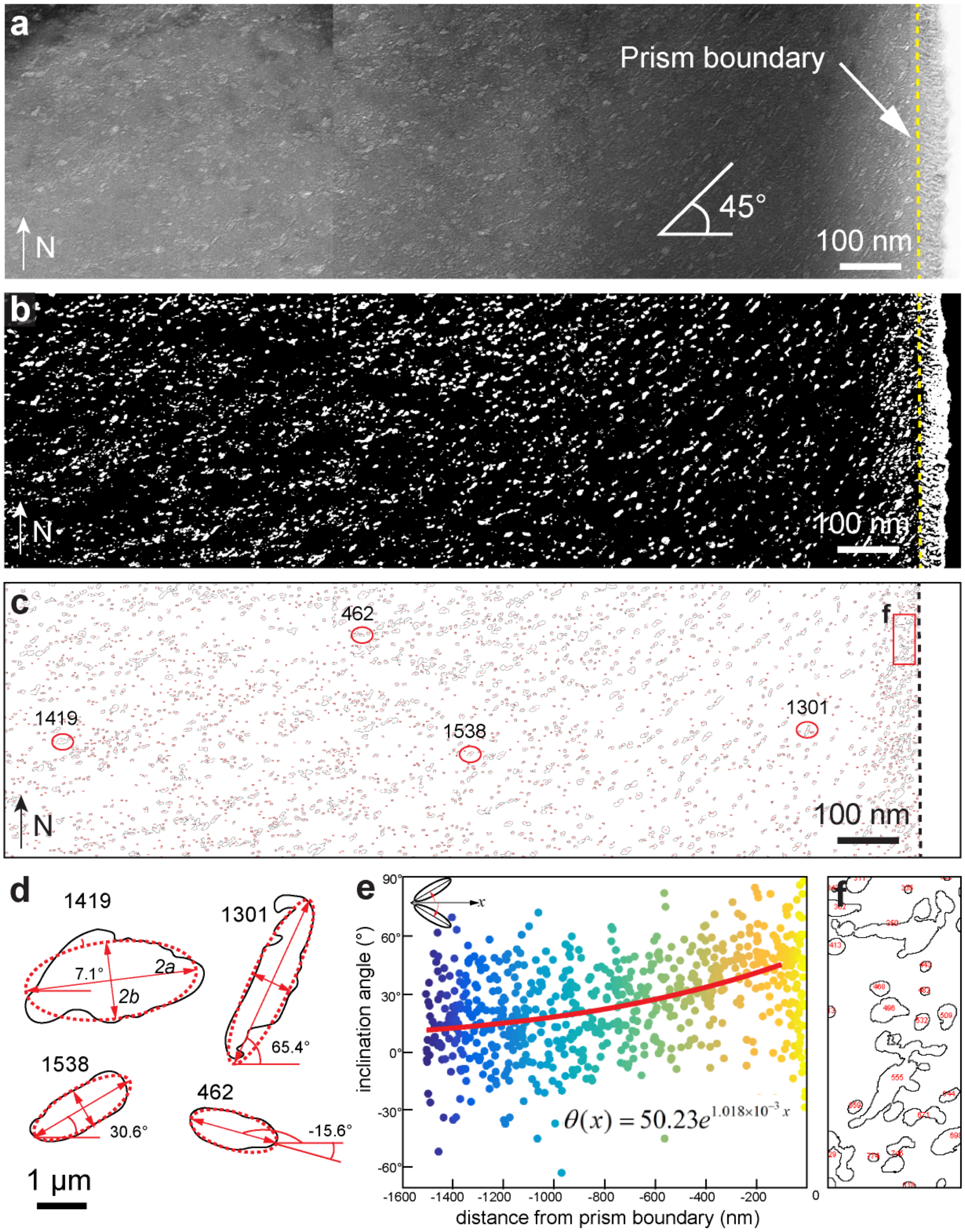

Supplementary Fig. 4. Orientation quantification of inclusions based on TEM imaging. (**a**) Stitched bright-field underfocused TEM images near the prism boundary. (**b**) Segmentation results using ilastik, where defect-like inclusions are shown as white particles. (**c**) Particle registration map using ImageJ, where each particle is registered and numbered separately. (**d**) Examples selected from (**c**) to demonstrate the ellipse fitting and orientations. (**e**) Inclusion orientation fitting based on particle registration results, where $x = 0$ marks the prism boundary. (**f**) Overlapping of high-density inclusions close to prism boundary.

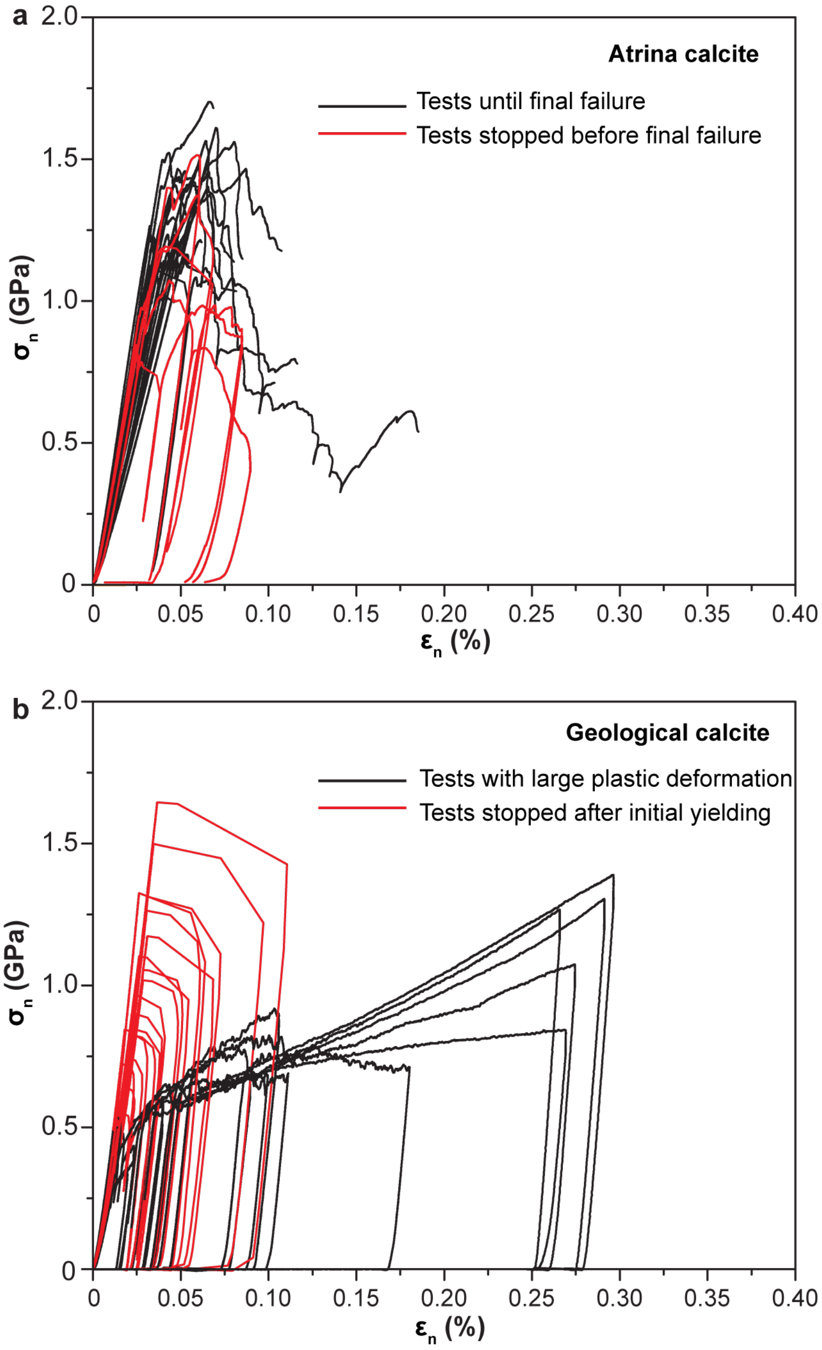

Supplementary Fig. 5. Engineering stress-strain (σ_n-ε_n) curves obtained from the micro-compression experiments on micro-pillars of (**a**) the biogenic calcite (n = 25) and (**b**) geological calcite (n = 22).

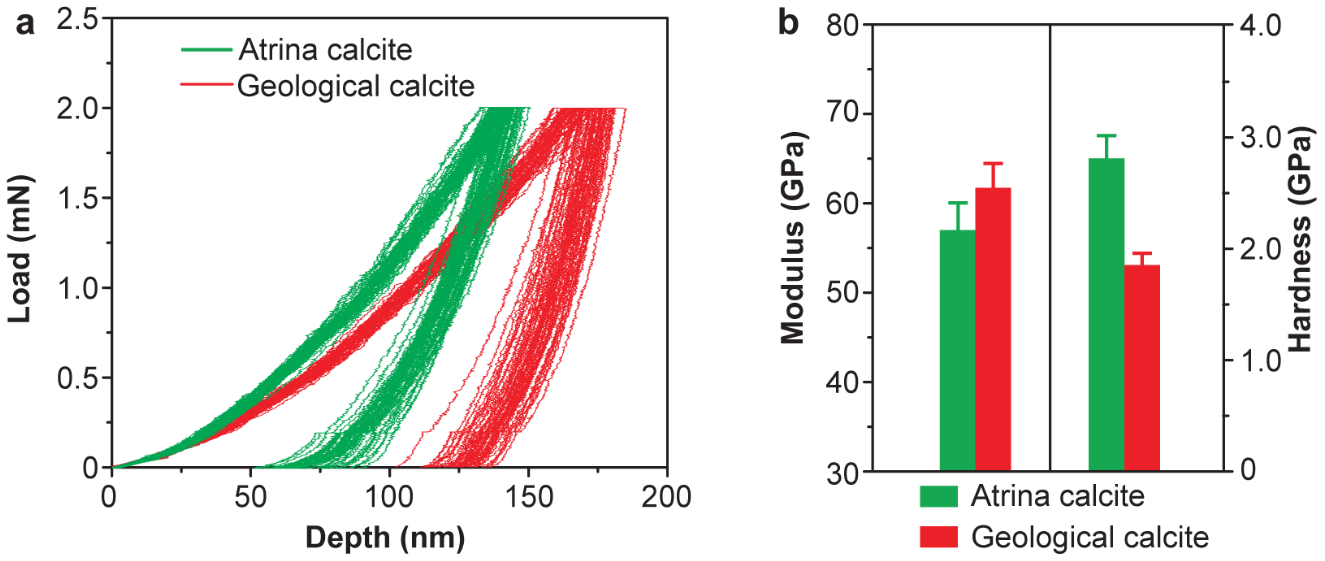

Supplementary Fig. 6. Instrumented nanoindentation results for the biogenic and geological calcite. (**a**) Nano-indentation load-depth curves. (**b**) Comparison of measured hardness and modulus.

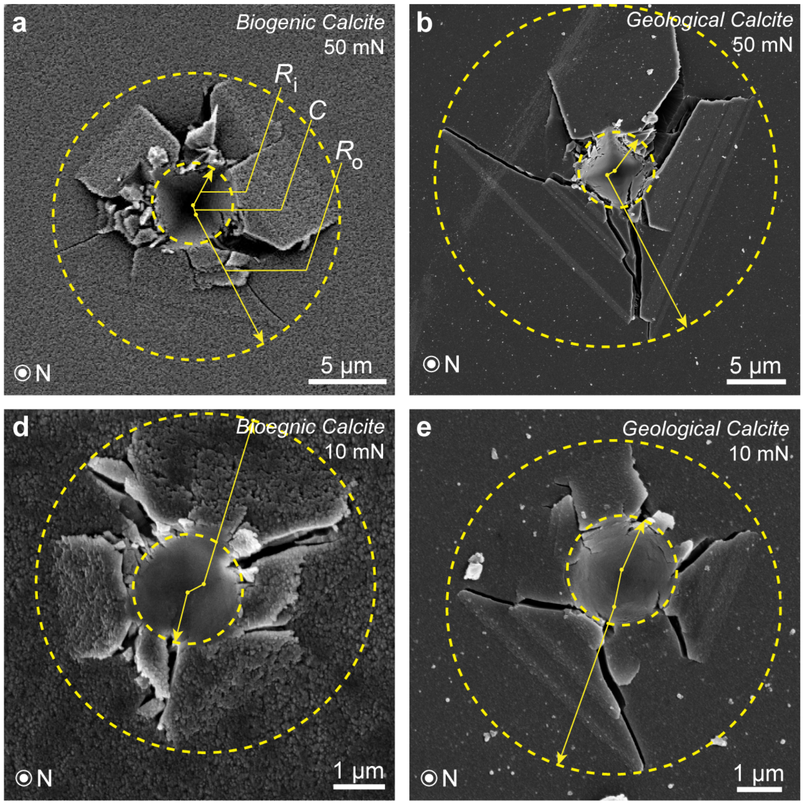

c

Micro-indentation 50 mN

	**Biogenic Calcite**	**Geological Calcite**
N	36	36
R_i	3.01 ± 0.31 μm	3.16 ± 0.23 μm
R_o	9.90 ± 0.87 μm	16.95 ± 2.87 μm
C	1.39 ± 0.78 μm	6.05 ± 2.59 μm

f

Micro-indentation 10 mN

	**Biogenic Calcite**	**Geological Calcite**
N	40	41
R^i	1.11 ± 0.03 μm	1.16 ± 0.06 μm
R^o	3.19 ± 0.33 μm	4.33 ± 0.91 μm
C	0.54 ± 0.33 μm	1.03 ± 0.77 μm

Supplementary Fig. 7. Fracture patterns of micro-indentations on the biogenic and geological calcite with a conospherical tip (tip radius ~ 1 μm; semi-angle 30°). Fracture patterns of the biogenic and geological calcite under maximum load of (**a,b**) 50 mN and (**d,e**) 10 mN, and (**c,f**) corresponding fracture pattern quantification, where R_i represents the radius of inner indentation crater, R_o denotes the radius of the entire fracture pattern by fitting it with smallest circle, and C corresponds to the distance between the centers of the two fitted circles.

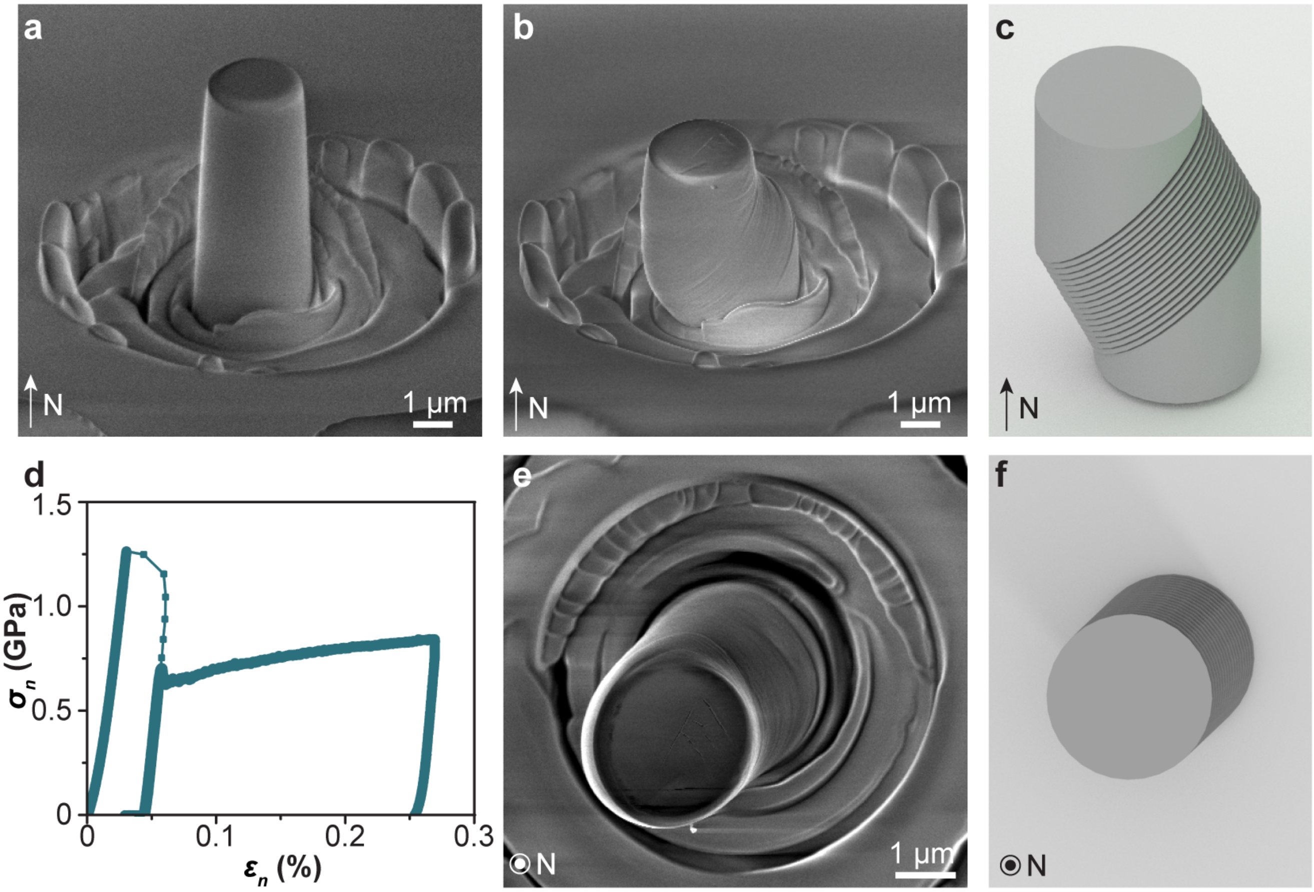

Supplementary Fig. 8. Observations of plastic deformation based on a single {10$\bar{1}$4} slip system during the compression of geological calcite micro-pillars SEM images of the micro-pillar (**a**) before and (**b**) after compression test. (**c**) 3D schematic illustration of one {10$\bar{1}$4} slip system activated in the micro-pillar. Here the actual slip angle was used for the 3D rendering, and the angle between {10$\bar{1}$4} planes and the c-axis is 44.6°. In addition, we maintained the same observation angle as the SEM imaging condition, where is 52° between the pillar axis and view angle. (**d**) The σ_n-ε_n curve corresponding to this compression test. (**e**) The top-view SEM image of the same micro-pillar after compression and **(f)** the corresponding top-view image of the 3D rendering.

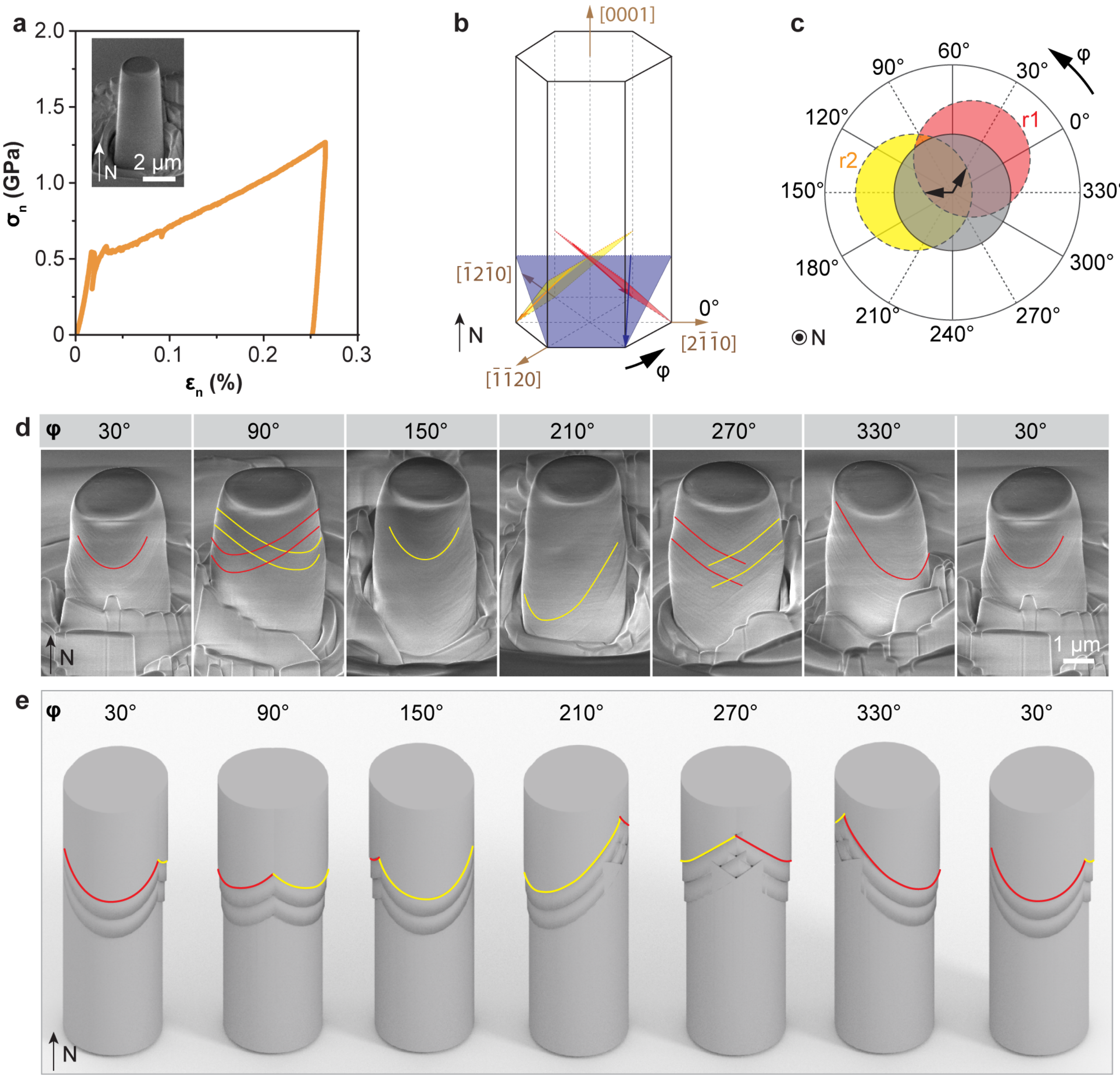

Supplementary Fig. 9. Observations of plastic deformation based on two {101̄4} slip systems during the compression of geological calcite micro-pillars. (**a**) The σ_n-ε_n curve for a representative test where the micro-pillar underwent a large plastic deformation (ε_n > 25%). Inset shows the corresponding micro-pillar before the compression test. (**b**) Schematic diagram of the crystallographic orientation and planes in calcite. Three {101̄4} slip planes are illustrated, i.e. r_1-(101̄4)[2̄021], r_2-(1̄104)[22̄01], and r_3-(01̄14)[022̄1] are shown in red, yellow, and blue, respectively. A polar angle, φ, is defined with respect to the direction [21̄1̄0], *i.e.*, *a* axis. In this notation, the slip orientation on planes r_1 and r_2 are in 30° and 150°, as shown in the top viewed schematic diagram in (**c**). (**d**) A series of SEM images of the compressed micro-pillar observed at φ of 30°, 90°, 150°, 210°, 270°, and 330° by rotating the stage. The slip lines for the two slip orientations, r_1 and r_2, are highlighted as the red and yellow lines, respectively. (**e**) A series of corresponding 3D schematic illustrations with r_1 and r_2 slip orientations highlighted. It shows an excellent match with the experimental observations in (**d**).

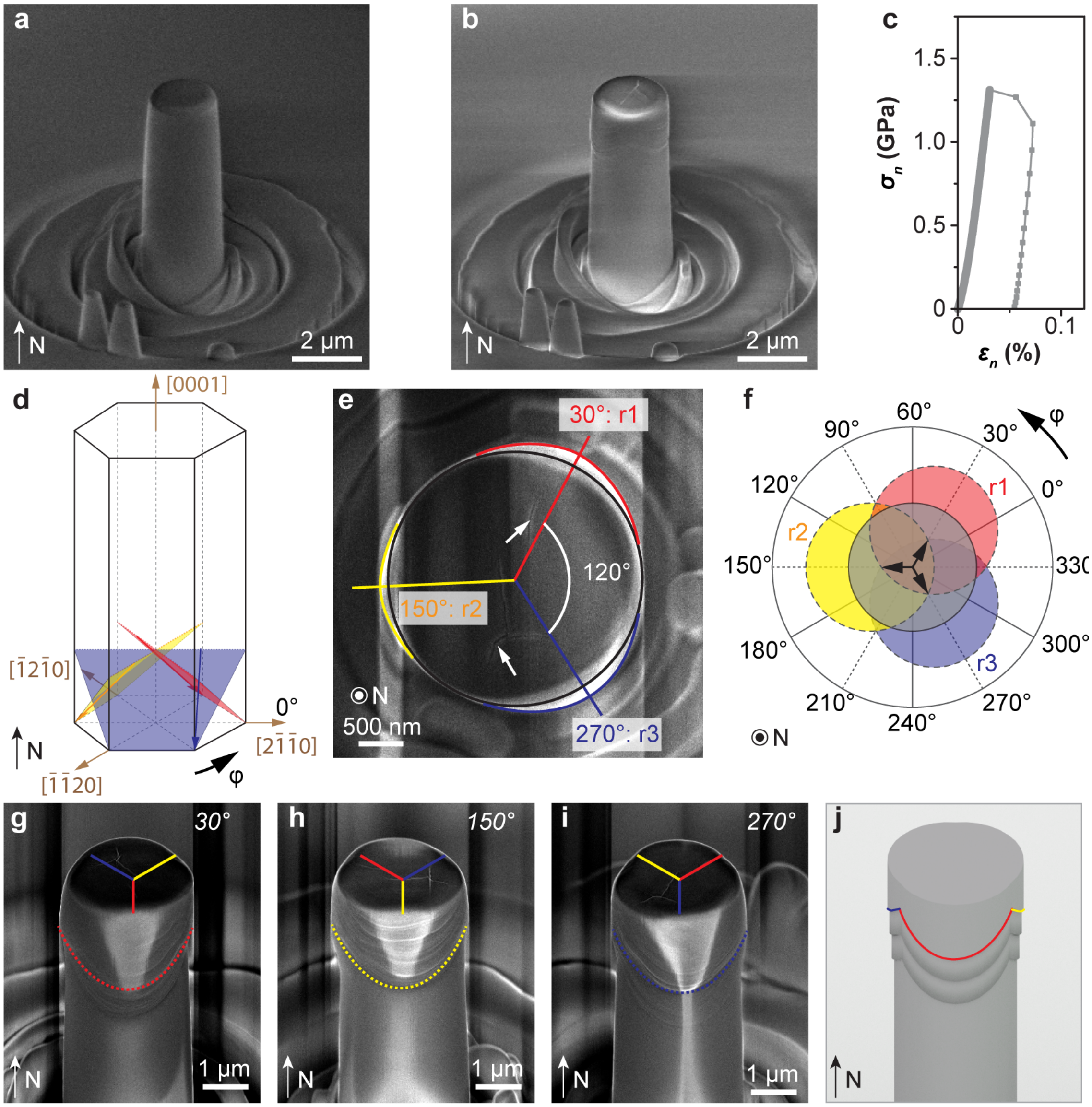

Supplementary Fig. 10. Observations of the localized plastic deformation at the top portion of a geological calcite micro-pillar after the uniaxial compression test, which was primarily based on three {$10\bar{1}4$} equivalent systems. SEM images of the micro-pillar (**a**) before and (**b**) after the compression test. (**c**) The engineering stress-strain curve corresponding to this measurement, showing that the compression test was stopped immediately after reaching the yield point. (**d**) Schematic diagram of the crystallographic orientation and planes. Three {$10\bar{1}4$} slip planes are illustrated, i.e. r_1-($10\bar{1}4$)[$\bar{2}021$], r_2-($\bar{1}104$)[$2\bar{2}01$], and r_3-($0\bar{1}14$)[$02\bar{2}1$] are shown in red, yellow, and blue planes, respectively. (**e**) A top-viewed SEM image of the deformed micro-pillar shows the three lobes of deformation resulted from the slip systems of r_1, r_2, and r_3. Note the three-fold symmetry of this deformation, consistent with a top-viewed schematic diagram in (**f**). (**g-i**) A series of SEM images of the compressed micro-pillar observed at φ of 30°,150°, and 270° by rotating the stage. The slip lines for r_1, r_2, and r_3 are highlighted by the red, yellow, and blue lines, respectively. Note that slip lines are identical in these three orientations because of their crystallographic equivalency. (**j**) A corresponding 3D schematic diagram of the slip systems.

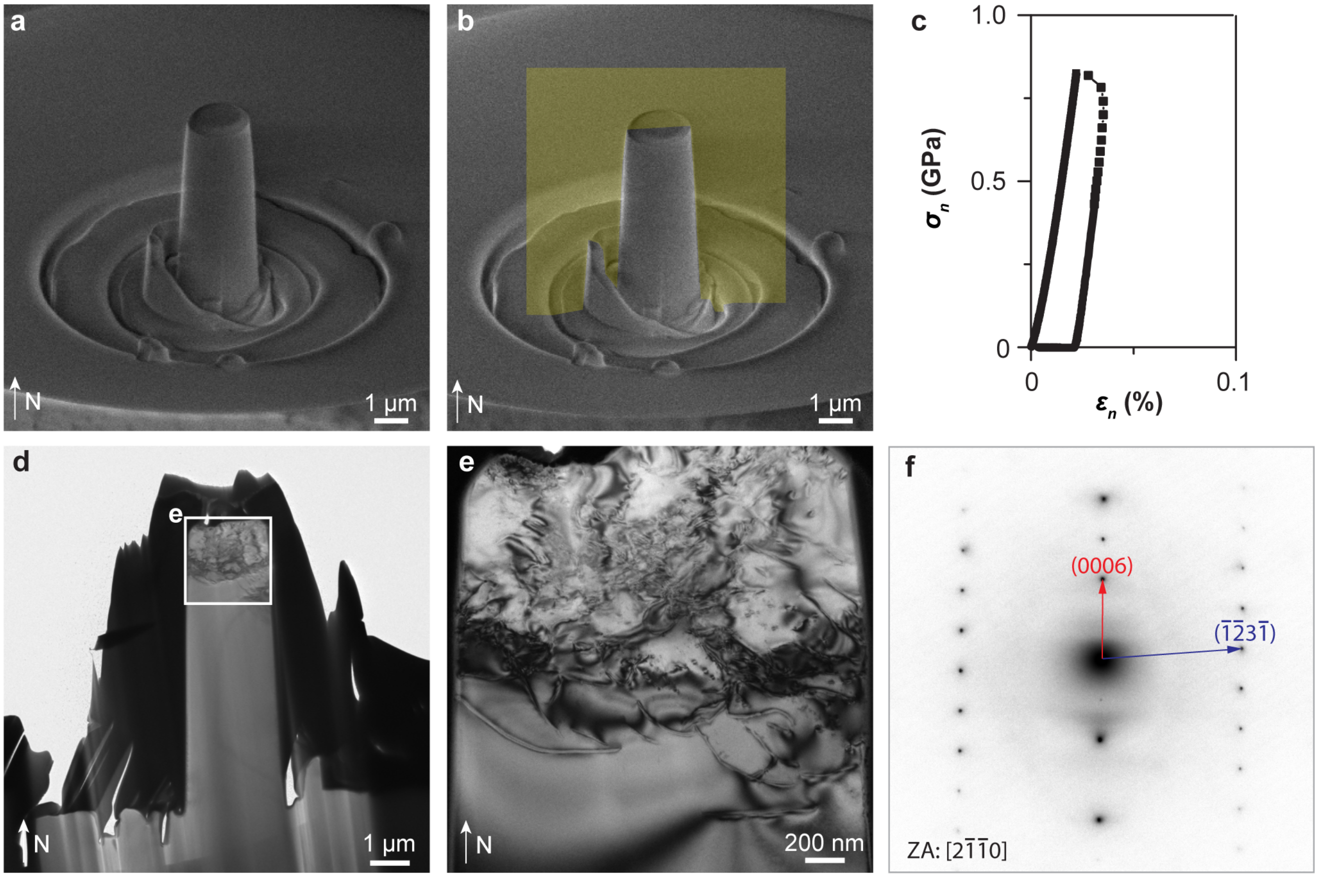

Supplementary Fig. 11. Localized plastic deformation via dislocations in the geological calcite micro-pillars immediately after yielding. SEM images of a micro-pillar (**a**) before and (**b**) after the compression test stopped immediately after yielding. The yellow plane in (**b**) represents the orientation and location for the TEM sample prepared via FIB milling. (**c**) The engineering stress-strain curve corresponding to this measurement, showing that the compression test was stopped immediately after the yield point was reached. (**d**) Bright-field TEM image of the deformed micro-pillar after the compression tests, showing that the deformation was localized at the top portion of the micro-pillar. (**e**) A TEM image acquired from the top deformed zone, showing the presence of dislocation arrays. (**f**) An SAED pattern taken from the undeformed region at the bottom portion of the micro-pillar, indicating its crystallographic *c* axis is aligned along the pillar axis.

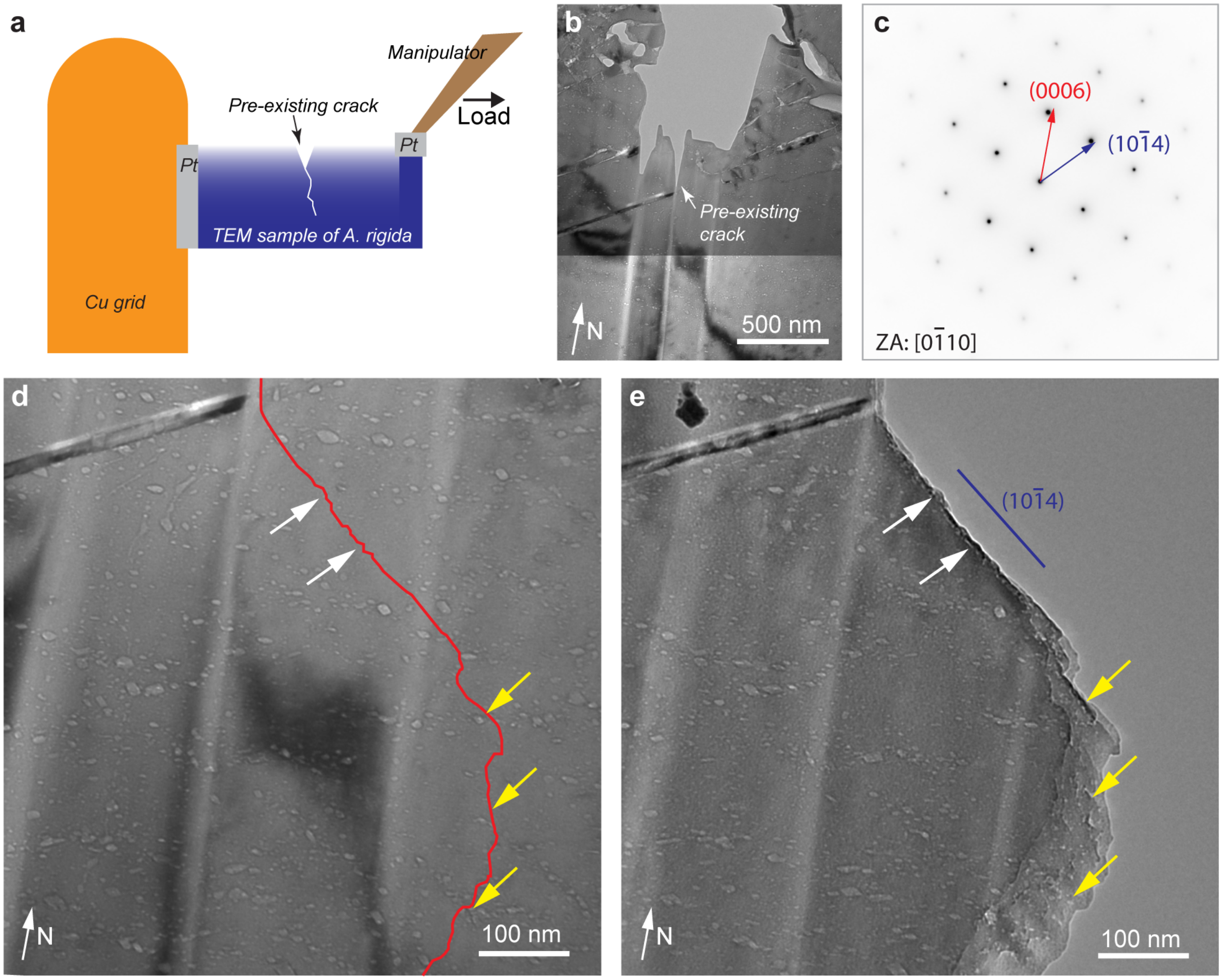

Supplementary Fig. 12. Fracture test on TEM thin sections of the biogenic calcite. (**a**) Schematic diagram showing the experiment setup. (**b**) TEM image of the original TEM sample before fracture, and the pre-existing crack is indicated by the white arrow. (**c**) Corresponding SAED pattern with zone axis of [0$\bar{1}$10]. Bright-field TEM image of the same region (**d**) before and (**e**) after fracture. The red line in (**d**) illustrates the crack observed in (**e**). Note that the nearly straight fracture line is close to the plane direction of (10$\bar{1}$4) cleavage plane. This suggests that the fracture in the regions with low density of defects of biogenic calcite can be similar to the cleavage behavior of pure calcite crystal (white arrows). In contrast, in the regions with high density of intracrystalline inclusions (yellow arrows), the cracks tended to be deflected by intracrystalline defects, forming a tortuous fracture path.

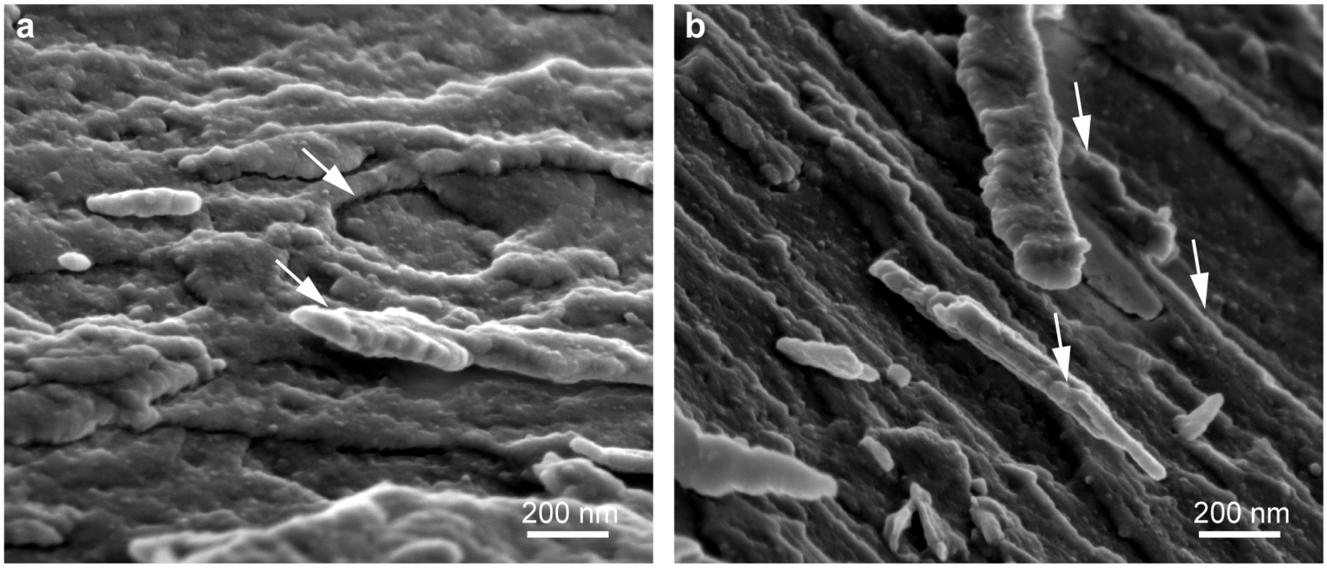

Supplementary Fig. 13. SEM observations of layered microstructures from fractured surfaces of the biogenic calcite (white arrows).

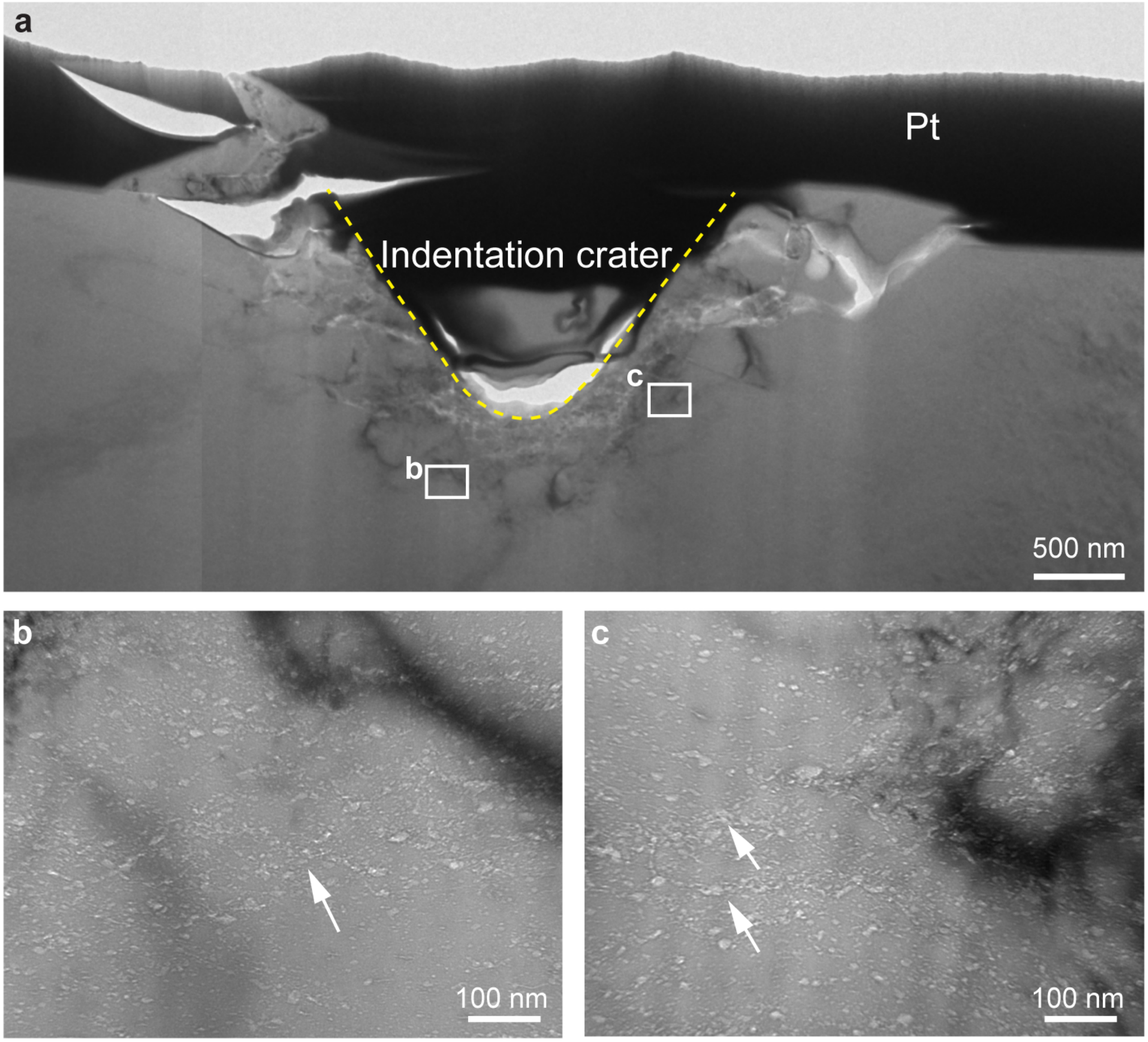

Supplementary Fig. 14. Cross-sectional TEM images of an indentation residue on the biogenic calcite. (**a**) Damage and fracture localization near the indentation crater; (**b**) and (**c**) cracks initiate and bridge adjacent defects (inclusions) at regions with high-density inclusions (white arrows).

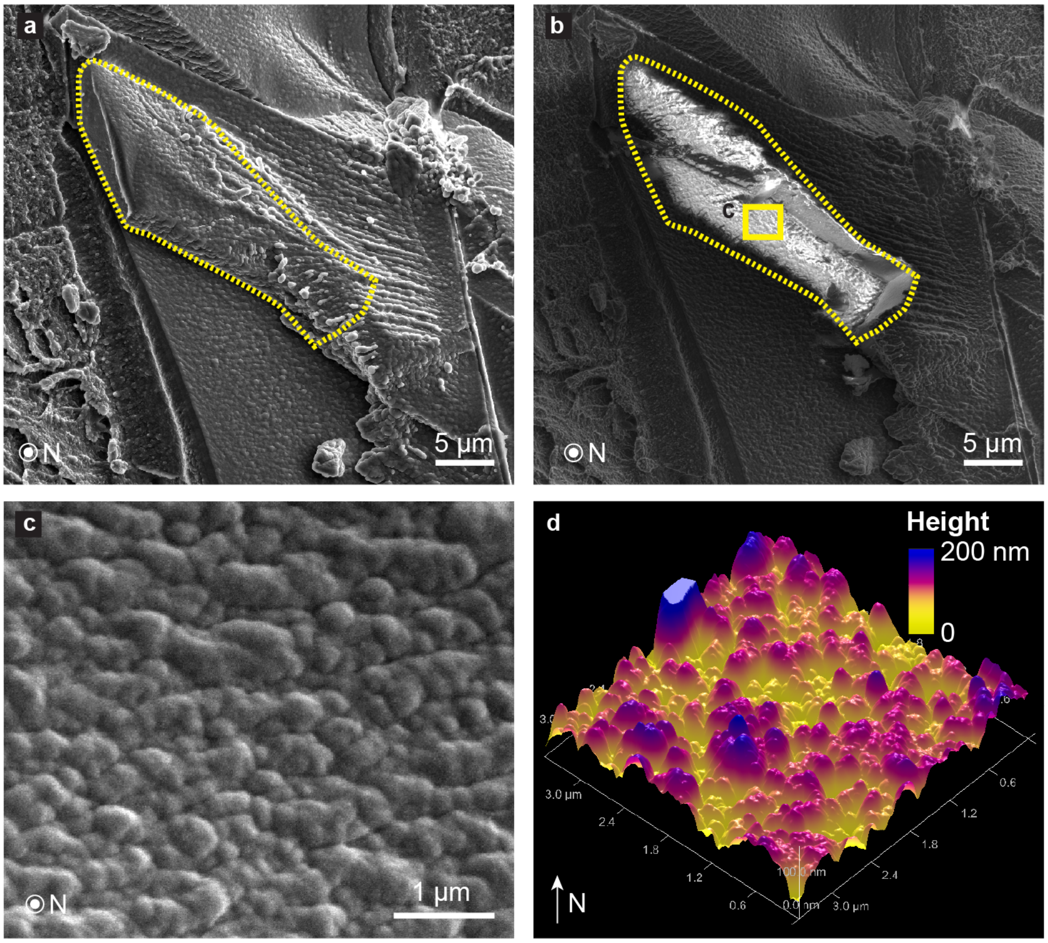

Supplementary Fig. 15. *In-situ* SEM fracture test on the biogenic calcite. (**a**) SEM images of the original structure. The yellow-dotted line contours out the region that was removed by using a micro-manipulator inside the SEM instrument. The as-resulted structure is shown in (**b**). (**c**) High-magnification SEM image of the freshly fractured surface. (**d**) AFM height profile image of the fractured surface. Both (**c**) and (**d**) illustrate the nanoscale roughness in the fractured surface.

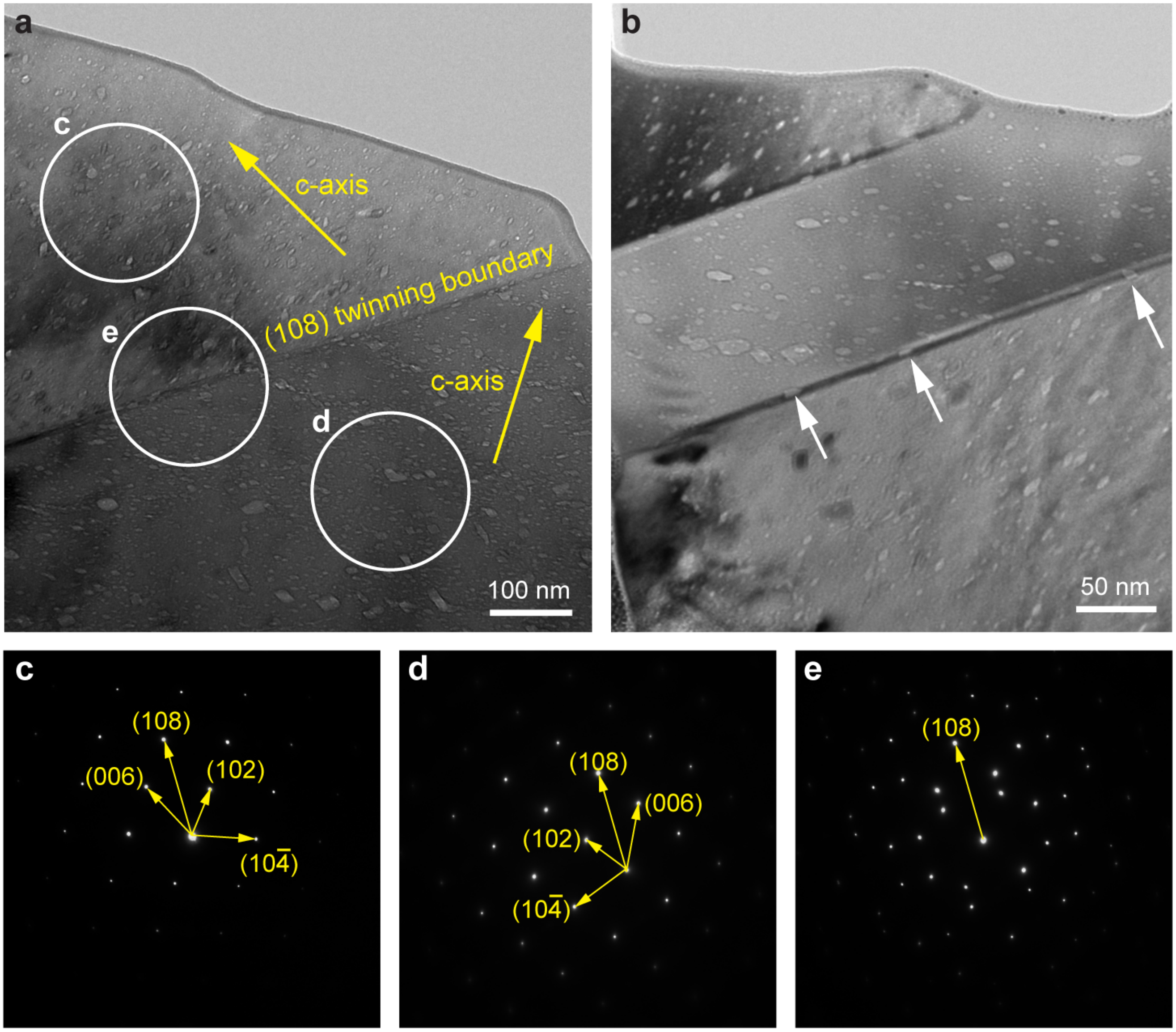

Supplementary Fig. 16. Deformation twinning in the biogenic calcite. (**a**,**b**) TEM images on the vertical cross-sections of the biogenic calcite after indentation tests showing twinning bands. The twinning boundaries pass through intracrystalline defects, which indicates that the twinning formation is not prohibited by the presence of intracrystalline defects (white arrows in **b**). (**c**-**e**) Corresponding SAED patterns acquired from the selected regions shown in (**a**), demonstrating the symmetric diffraction patterns due to twinning.

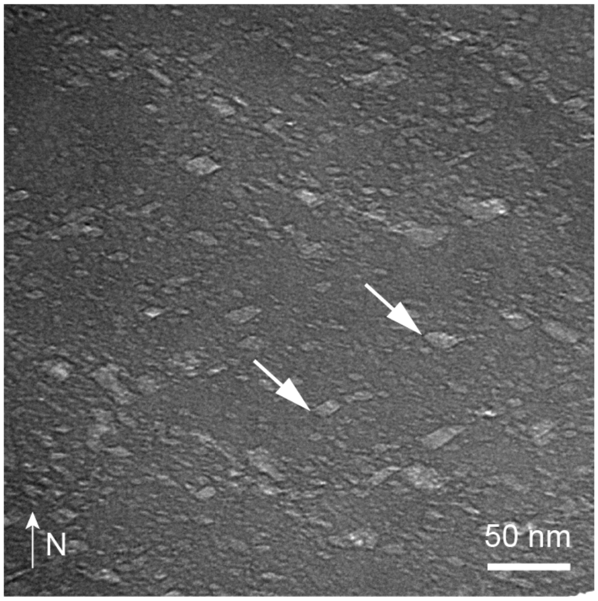

Supplementary Fig. 17. Intracrystalline defects with sharp corners. TEM images on the vertical cross-section of *A. rigida* showing defect-like inclusions with sharp corners (white arrows).

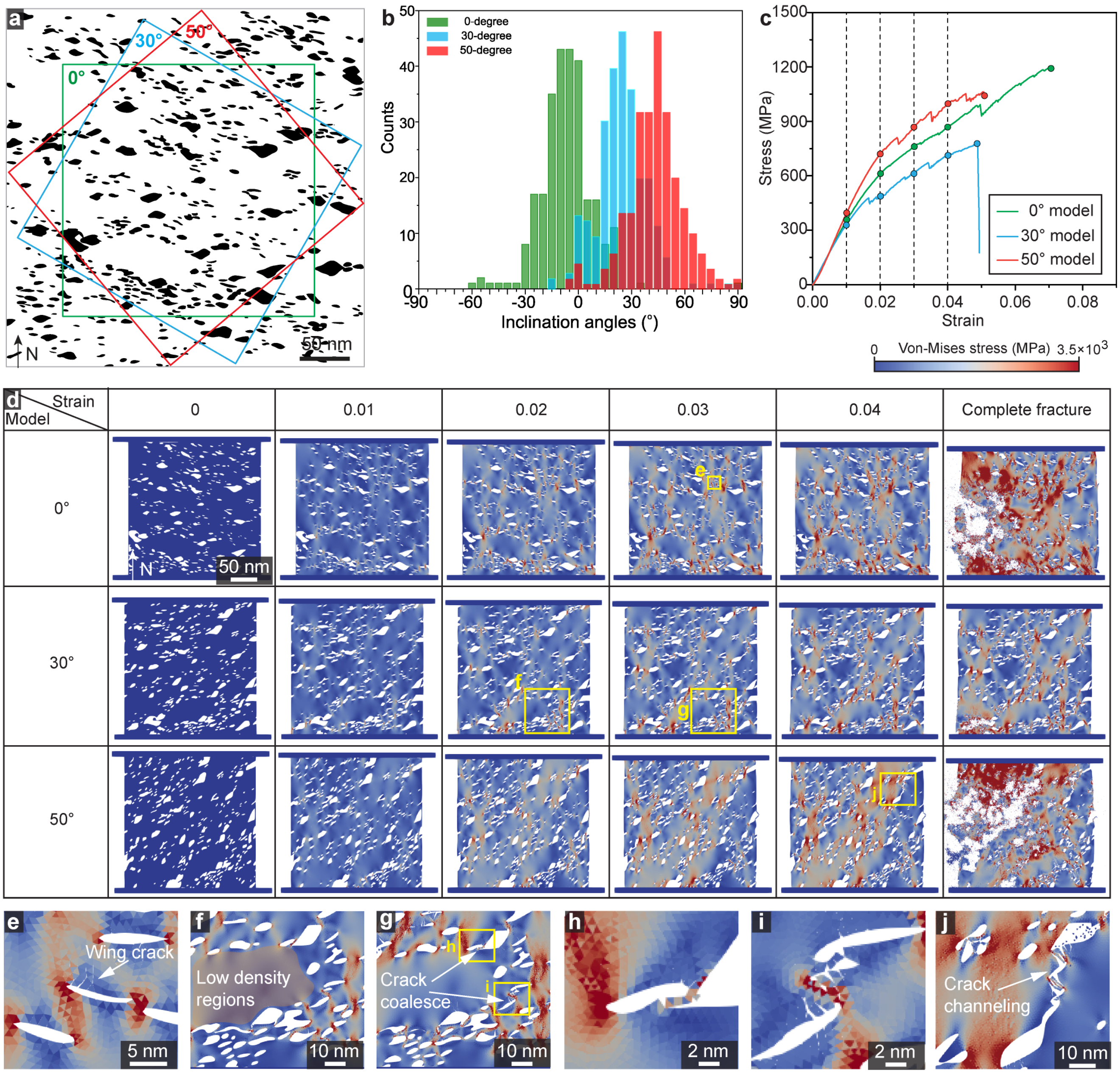

Supplementary Fig. 18. Fracture behavior of the biogenic calcite via FE/DE simulations. (**a**) 2D models with defects of different inclination angles, obtained by rotating the segmentation pattern from TEM image of longitudinal cross section of *A. rigida*. The nominal inclination angles for the 2D models are 0°, 30°, and 50°, respectively. (**b**) Frequency histogram of inclination angles of defects within each model, showing close match with the rotation angles. (**c**) Stress-strain curves for three models obtained from fracture simulations. (**d**) Typical crack evolution process in each model. (**e-j**) Close-up images at crack initiation sites, showing (**e**) formation of wing cracks, (**f-i**) coalescing crack development, and (**j**) crack channeling multiple defects with higher inclination angles.

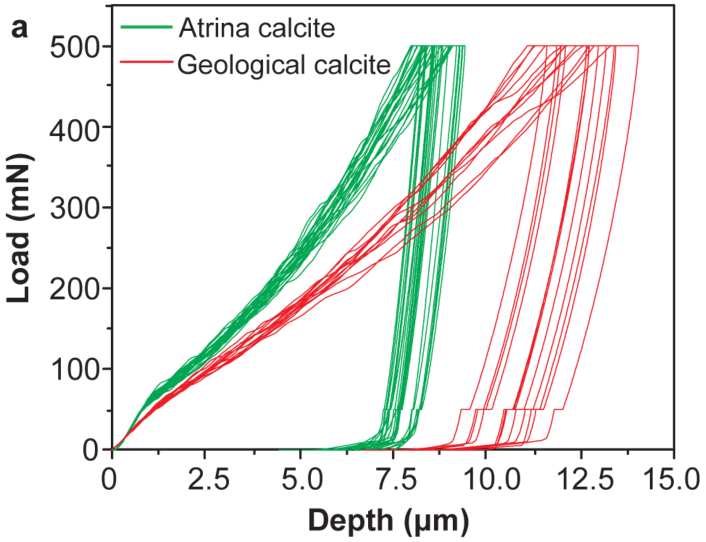

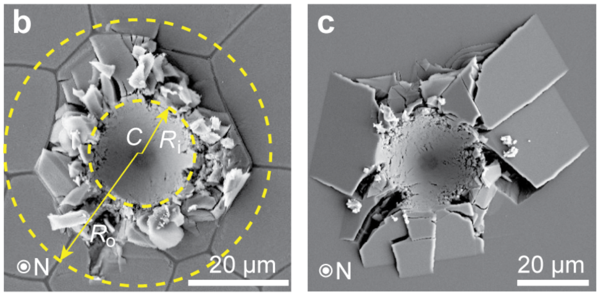

d **Micro-indentation 500 mN**

	Atirna Calcite	Geological calcite
N	23	12
***R**$_i$*	23.76 ± 1.88 μm	26.64 ± 2.45 μm
***R**$_o$*	53.24 ± 6.25 μm	128.54 ± 35.39 μm
C	2.30 ± 1.33 μm	22.36 ± 14.57 μm

Supplementary Fig. 19. High load indentations on the biogenic and geological calcite (500mN, conospherical tip radius ~ 5 µm; semi-angle 45°). (**a**) Load-depth curves of the biogenic and geological calcite. (**b**,**c**) SEM images on the representative fracture patterns of the biogenic and geological calcite, respectively. (**d**) Comparison of fracture pattern quantification in the biogenic and geological calcite, where R_i represents the radius of inner indentation crater, R_o denotes the radius of the entire fracture pattern by fitting it with smallest circle, and C corresponds to the distance between the centers of the two fitted circles.

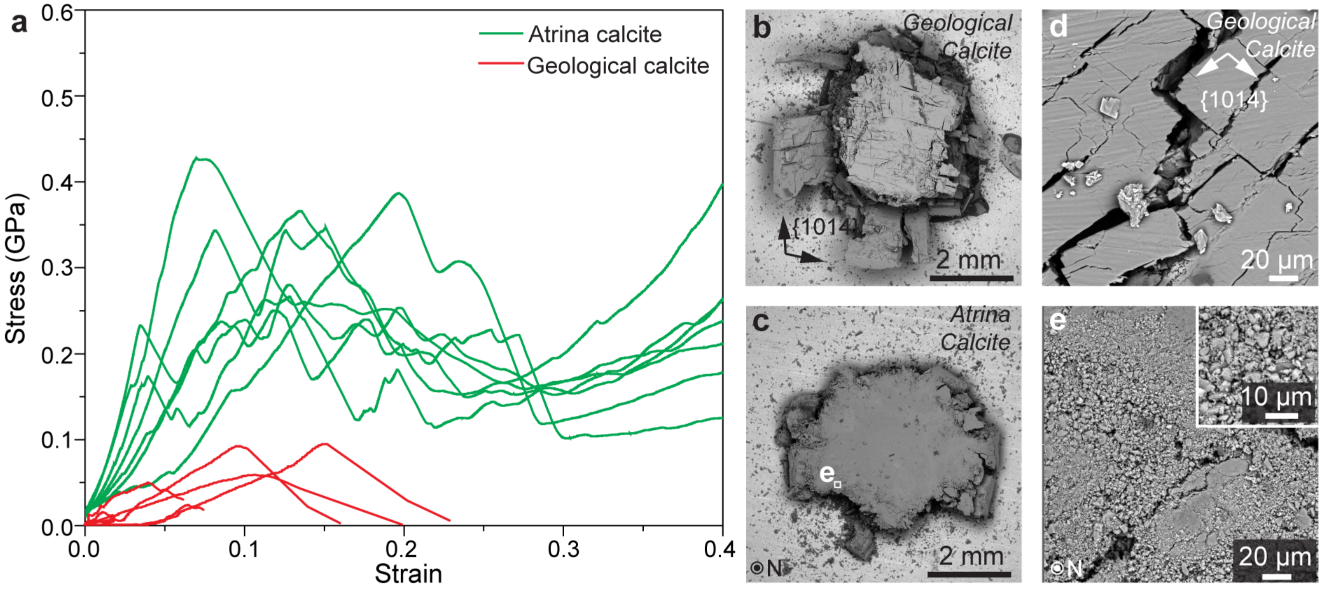

Supplementary Fig. 20. Macroscopic compression on the biogenic and geological calcite. (**a**) Stress-strain curves showing that the biogenic calcite has much higher strength than geological calcite while sustaining load beyond yield point. (**b,c**) Overview and (**d,e**) high magnification SEM images of the (**b,d**) geological and (**c,e**) biogenic calcite after macroscopic compression. The geological calcite deforms through large-scale cracking and fractures into large pieces, while the biogenic calcite in *A. rigida* fractures into micro-/nano-scale pieces (Inset of **e**).

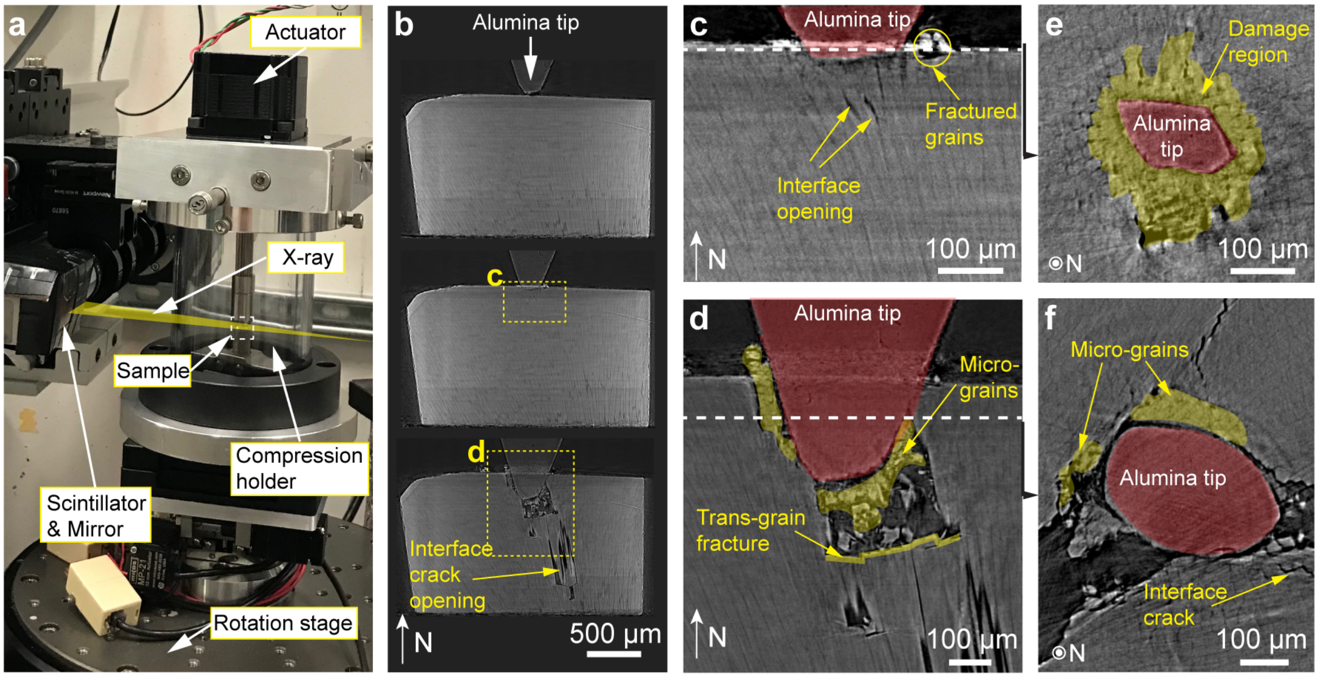

Supplementary Fig. 21. In-situ indentation tests on the biogenic calcite. (**a**) *In-situ* test setup with sample holder mounted on the rotation stage. (**b**) Selected reconstruction slices along from *in-situ* indentation tests with an alumina tip on *A. rigida* samples. Scans were obtained between each load step to capture the deformation process the deformation and crack propagation. (**c,d**) High magnification vertical slices from *in-situ* data showing different deformation mechanisms of the prismatic layer in biogenic calcite. (**e,f**) Horizontal slices corresponding to the latitude of dashed white lines in (**c,d**), respectively.

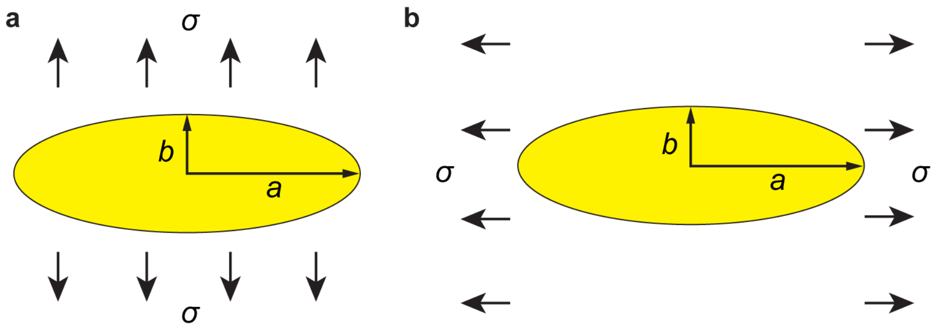

Supplementary Fig. 22. Stress intensity factors of crack propagating along (**a**) long and (**b**) short axes of an elliptical defect.

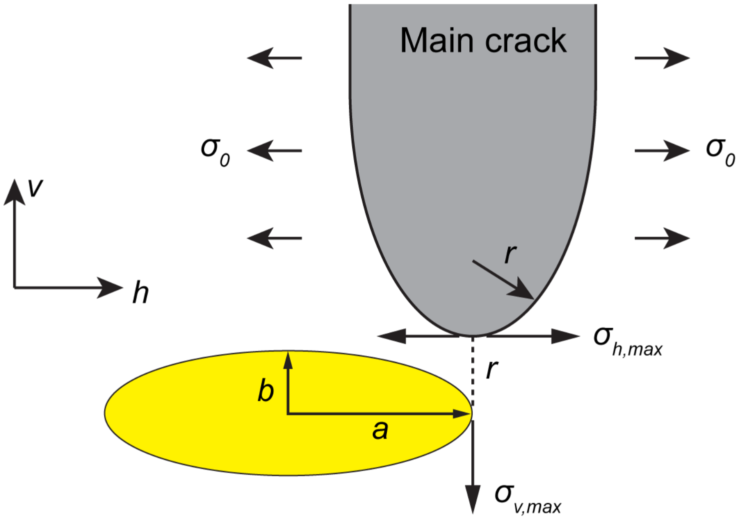

Supplementary Fig. 23. Stress intensity factor of an elliptical defect deflecting vertical crack into horizontal direction.

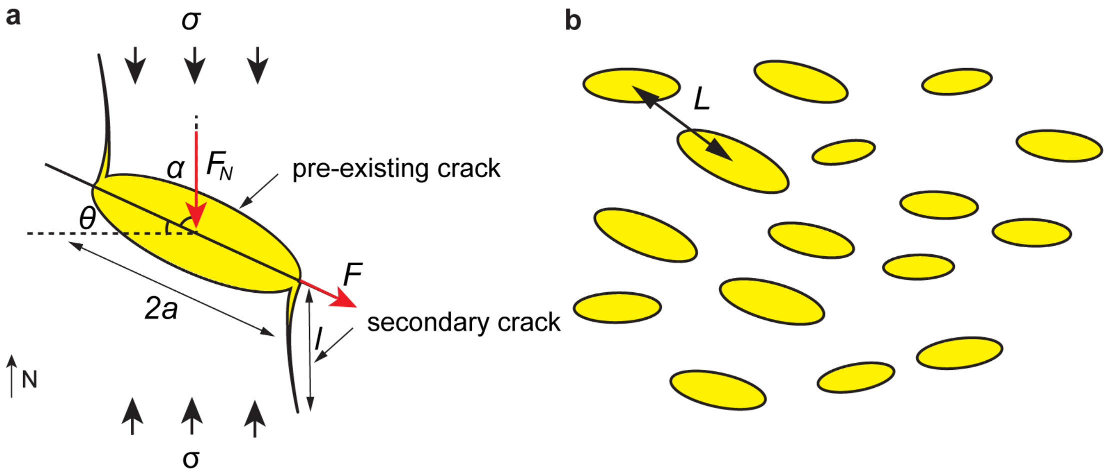

Supplementary Fig. 24. Mode I crack opening models under far field compressive stress of (**a**) a single non-interacting defect, and (**b**) multiple interacting defects with average spacing of *L*.

Supplementary references

1. Nudelman, F., Chen, H. H., Goldberg, H. a., Weiner, S. & Addadi, L. Spiers Memorial Lecture : Lessons from biomineralization: comparing the growth strategies of mollusc shell prismatic and nacreous layers in *Atrina rigida*. *Faraday Discuss.* **136**, 9–25 (2007).
2. De Bresser, J. H. P. & Spiers, C. J. Slip systems in calcite single crystals deformed at 300-800°C. *J. Geophys. Res.* **98**, 6397–6409 (1993).
3. De Bresser, J. H. P. & Spiers, C. J. Strength characteristics of the r, f, and c slip systems in calcite. *Tectonophysics* **272**, 1–23 (1997).
4. Barber, D. J., Wenk, H.-R., Hirth, G. & Kolstedt, D. L. Dislocations in Minerals. in *Dislocations in Solids* vol. 16 (Elsevier B.V., 2010).
5. Cheng, Y. T. & Cheng, C. M. Scaling approach to conical indentation in elastic-plastic solids with work hardening. *J. Appl. Phys.* **84**, 1284–1291 (1998).
6. Kim, Y. Y. *et al.* Tuning hardness in calcite by incorporation of amino acids. *Nat. Mater.* **15**, 903–910 (2016).
7. Kunitake, M. E., Mangano, L. M., Peloquin, J. M., Baker, S. P. & Estroff, L. A. Evaluation of strengthening mechanisms in calcite single crystals from mollusk shells. *Acta Biomater.* **9**, 5353–5359 (2013).
8. Cook, J. & Gordon, J. E. A mechanism for the control of crack propagation in all-brittle systems. *Proc. R. Soc. London. Ser. A. Math. Phys. Sci.* **282**, 508–520 (1964).
9. Inglis, C. E. Stresses in a plate due to the presence of cracks and sharp corners. *Trans Inst Nav. Arch.* **55**, 219–241 (1913).
10. Germanovich, L. N., Salganik, R. L., Dyskin, A. V. & Lee, K. K. Mechanisms of brittle fracture of rock with pre-existing cracks in compression. *Pure Appl. Geophys.* **143**, 117–149 (1994).
11. Callister, W. D. in *Materials Science and Engineering: An introduction* (Von Hoffmann Press, 5th Editio., 1999), pp. 381–420.
12. Diaz-Rubio, F. G., Perez, J. R. & Galvez, V. S. The spalling of long bars as reliable method of measuring the dynamic tensile strength of ceramics. *Int. J. Impact Eng.* **27**, 161–177 (2002).
13. Tada, H., Paris, P. C. & Irwin, G. R. *The Stress Analysis of Cracks Handbook* (Third Edit., 2000).
14. Barthelat, F. Designing nacre-like materials for simultaneous stiffness, strength and toughness: Optimum materials, composition, microstructure and size. *J. Mech. Phys. Solids.* **73**, 22–37 (2014).
15. Lin, A. Y. M. & Meyers, M. A. Interfacial shear strength in abalone nacre. *J. Mech. Behav. Biomed. Mater.* **2**, 607–612 (2009).
16. Mahabadi, O. K., Lisjak, A., Munjiza, A. & Grasselli, G. Y-Geo: New combined finite-discrete element numerical code for geomechanical applications. *Int. J. Geomech.* **12**, 676–688 (2012).
17. Lisjak, A. & Grasselli, G. A review of discrete modeling techniques for fracturing processes in discontinuous rock masses. *J. Rock Mech. Geotech. Eng.* **6**, 301–314 (2014).
18. Robach, J. S., Stock, S. R. & Veis, A. Transmission electron microscopy characterization of macromolecular domain cavities and microstructure of single-crystal calcite tooth plates of the sea urchin Lytechinus variegatus. *J. Struct. Biol.* **151**, 18–29 (2005).
